# Physical activity and the risk of heart failure: a systematic review and dose–response meta-analysis of prospective studies

**DOI:** 10.1007/s10654-020-00693-6

**Published:** 2020-12-17

**Authors:** Dagfinn Aune, Sabrina Schlesinger, Michael F. Leitzmann, Serena Tonstad, Teresa Norat, Elio Riboli, Lars J. Vatten

**Affiliations:** 1 Department of Epidemiology and Biostatistics, School of Public Health, Imperial College London, St. Mary’s Campus, Norfolk Place, Paddington, London, W2 1PG UK; 2 Department of Nutrition, Bjørknes University College, Oslo, Norway; 3 Department of Endocrinology, Morbid Obesity and Preventive Medicine, Oslo University Hospital Ullevål, Oslo, Norway; 4 Institute for Biometry and Epidemiology, German Diabetes Center, Leibniz Institute for Diabetes Research at the Heinrich-Heine-University Düsseldorf, Düsseldorf, Germany; 5 Department of Epidemiology and Preventive Medicine, Regensburg University Medical Center, Regensburg, Germany; 6 Department of Public Health and Nursing, Faculty of Medicine, Norwegian University of Science and Technology, Trondheim, Norway

**Keywords:** Physical activity, Walking, Cardiorespiratory fitness, Heart failure, Meta-analysis

## Abstract

Although physical activity is an established protective factor for cardiovascular diseases such as ischemic heart disease and stroke, less is known with regard to the association between specific domains of physical activity and heart failure, as well as the association between cardiorespiratory fitness and heart failure. We conducted a systematic review and meta-analysis of prospective observational studies to clarify the relations of total physical activity, domains of physical activity and cardiorespiratory fitness to risk of heart failure. PubMed and Embase databases were searched up to January 14th, 2020. Summary relative risks (RRs) were calculated using random effects models. Twenty-nine prospective studies (36 publications) were included in the review. The summary RRs for high versus low levels were 0.77 (95% CI 0.70–0.85, I^2^ = 49%, n = 7) for total physical activity, 0.74 (95% CI 0.68–0.81, I^2^ = 88.1%, n = 16) for leisure-time activity, 0.66 (95% CI 0.59–0.74, I^2^ = 0%, n = 2) for vigorous activity, 0.81 (95% CI 0.69–0.94, I^2^ = 86%, n = 3) for walking and bicycling combined, 0.90 (95% CI 0.86–0.95, I^2^ = 0%, n = 3) for occupational activity, and 0.31 (95% CI 0.19–0.49, I^2^ = 96%, n = 6) for cardiorespiratory fitness. In dose–response analyses, the summary RRs were 0.89 (95% CI 0.83–0.95, I^2^ = 67%, n = 4) per 20 MET-hours per day of total activity and 0.71 (95% CI 0.65–0.78, I^2^ = 85%, n = 11) per 20 MET-hours per week of leisure-time activity. Nonlinear associations were observed in both analyses with a flattening of the dose–response curve at 15–20 MET-hours/week for leisure-time activity. These findings suggest that high levels of total physical activity, leisure-time activity, vigorous activity, occupational activity, walking and bicycling combined and cardiorespiratory fitness are associated with reduced risk of developing heart failure.

**Electronic supplementary material:**

The online version of this article (10.1007/s10654-020-00693-6) contains supplementary material, which is available to authorized users.

## Introduction

Cardiovascular disease is the leading cause of death globally, accounting for 17.9 million deaths in 2015 [1]. In the U.S., heart failure affected approximately 5 million persons in 2005, and economic costs were estimated at 27.9 billion US dollars [2]. Mortality in patients with heart failure remains high, ranging from 20 to 40% despite advances in the management of the disease [3, 4]. Established or suspected risk factors for heart failure include age, histories of coronary heart disease, valvular heart disease, left ventricular hypertrophy, atrial fibrillation, hypertension, family history of cardiovascular disease, diabetes mellitus, high heart rate, smoking, general and abdominal adiposity, and low physical activity [5–10].

Although a substantial amount of data has consistently shown that physical activity reduces the risks of coronary heart disease [11] and stroke [11], fewer studies have been published on the association between physical activity and the risk of heart failure [12–38]. Although most studies have shown reduced risk of heart failure with higher physical activity [12–19, 21–25, 27, 28, 30, 31, 34, 35], other studies have found either no association [29, 32], an inverse association among women but not men [20, 33], or a U-shaped association [26]. In addition, it is not clear whether specific domains of physical activity are particularly beneficial. Some studies [14, 16, 18, 19, 22, 35, 39] found a reduced risk of heart failure with high total activity, while other studies found no significant association [21, 26, 29, 32]. All [12, 15, 17, 20–23, 26–28, 33] but one [24] study on leisure-time activity reported inverse associations, two studies found inverse associations for vigorous activity [13, 31], three [22, 26, 40] of four [20, 22, 26, 40] studies on walking reported inverse associations, and one [15] of three [15, 22, 26] studies on occupational activity reported inverse associations with heart failure. Some studies on leisure-time activity and heart failure reported results stratified by ethnicity [16, 19, 28, 39], and three [16, 19, 39] of four [16, 19, 28, 39] studies found inverse associations in Caucasians, two [16, 39] of four [16, 19, 28, 39] studies found inverse associations in African Americans, two [19, 28] of three [19, 28, 39] studies found inverse associations among Hispanics, and one [19] of two [19, 28] studies found inverse associations in Asians. All available studies on cardiorespiratory fitness reported inverse associations with heart failure, but the magnitude of the risk reductions varied considerably from 45 to 84% decreases in risk [35, 41–47].

Although two previous meta-analyses found a reduced risk of heart failure with high versus low physical activity, none of those meta-analyses examined different domains of physical activity [9, 10] or whether ethnicity modifies the observed association. In addition, at least 18 additional studies (19 publications) [23–35, 37–39, 42–45, 47] on physical activity or cardiorespiratory fitness and risk of heart failure with more than 82,000 cases among > 3.6 million participants have since been published. A more up-to-date summary of the evidence regarding physical activity and domains of physical activity and cardiorespiratory fitness and risk of heart failure could also be useful for risk assessments, such as the Global Burden of Disease, which have not included data regarding physical activity and heart failure in their previous assessments [48].

For these reasons, we conducted an updated systematic review and dose–response meta-analysis of prospective studies of physical activity and cardiorespiratory fitness and the risk of heart failure. We aimed to clarify the strength of the association, the shape of the dose–response relationship, potential sources of heterogeneity between studies, differences by domains of activity and effect modification by ethnicity.

## Methods

### Search strategy and inclusion criteria

PubMed and Embase databases were searched up to January 14th 2020 for eligible studies. A list of search terms used is provided in the Supplementary Text. We followed standard criteria for reporting meta-analyses [49]. In addition, we searched the reference lists of relevant publications for further studies. Study quality was assessed using the Newcastle–Ottawa scale [50].

### Study selection

To be included, a study had to be a prospective cohort, case-cohort, or nested case–control study and to investigate the association between physical activity or cardiorespiratory fitness and risk of heart failure in adults from the general population. Studies in specific patient groups were excluded. Estimates of the relative risk (RR; hazard ratio, risk ratio, odds ratio) with 95% confidence intervals (CIs) adjusted for at least one confounding factor had to be available. For the dose–response meta-analysis, a quantitative measure of activity level and the total number of cases and person-years had to be reported. When multiple publications were available from the same study we used the study with the largest number of heart failure cases. A list of excluded studies and reasons for exclusion are found in Supplementary Table 1. Of the studies included in the review [12–47], two studies were not included in the meta-analyses because there was only one study on each exposure; changes in physical activity [37] and changes in cardiorespiratory fitness [47]. Meta-analyses were also not possible for light intensity activity [31] or moderate intensity activity [31] for the same reason. Three studies on different measures of physical activity (total leisure-time activity, walking, walking pace, and total physical activity) and heart failure mortality [36, 38, 40] were excluded from the primary analyses because some evidence suggests that physical activity may improve survival in heart failure patients [51], however, sensitivity analyses were conducted including these studies in the respective analyses. Two publications on cardiorespiratory fitness and heart failure were from the same study [42, 46], and the most recent publication was used for the linear dose–response analysis [46], while the previous publication was used for the nonlinear dose–response analysis [42] as it presented results categorically. Three publications on physical activity were also from the same study [23, 31, 39], and the most recent publication was included in the main analysis [31], however, the previous publications were included in subgroup analyses by ethnicity [39] and in analyses of physical activity recommendations [23]. Other publications that were from the same studies reported on different aspects of physical activity and were therefore included in the respective analyses [14, 15, 17, 25].

### Data extraction

We extracted the following data from each study: first author’s last name, publication year, country where the study was conducted, study period, sample size, number of cases, type of exposure, exposure level, RRs and 95% CIs for each category of physical activity and cardiorespiratory fitness, and variables adjusted for in the analysis. Data were extracted by one reviewer (DA) and checked for accuracy by a second reviewer (SS).

### Statistical analysis

Random effects models were used to calculate summary RRs and 95% CIs for the highest versus lowest level of physical activity and for the dose–response analysis [52]. The average of the natural logarithm of the RRs was estimated and the RR from each study was weighted using random effects weights. A two-tailed *p* < 0.05 was considered statistically significant. When studies reported separate but not combined results for men and women or other subgroups, the subgroup-specific results were combined using a fixed-effects model to obtain an overall estimate which was used for the main analysis. For studies using the highest category of physical activity or cardiorespiratory fitness as the reference category, we recalculated the RRs such that the lowest category became the reference category using the method by Hamling [53].

For the dose–response meta-analysis, we computed study-specific slopes (linear trends) and 95% CIs from the natural logs of the RRs and CIs across categories of physical activity or fitness using the method of Greenland and Longnecker [54]. That method requires that the distribution of cases and person-years or non-cases and the RRs with the variance estimates for at least three quantitative exposure categories are known. When the distribution of cases or person-years was not reported, we estimated an approximate distribution using the total number of cases/person-years using a method previously described [55]. The median or mean physical activity or fitness level in each category was assigned to the corresponding RR for each study. For studies that reported ranges of activity or fitness, we estimated the midpoint for each category by calculating the average of the lower and upper bounds. When the highest or lowest category was open-ended, we assumed the open-ended interval length to be the same as the adjacent interval. For studies that reported physical activity by frequency per week or month, we converted the frequencies to hours per week or month by assigning a dose of 45 min per session [56] and for one study on vigorous activity, we further converted the results to MET-hours/week by multiplying number of hours/week by a factor of 8 [11]. A potential nonlinear dose–response relationship between physical activity and heart failure was examined using restricted cubic splines with 3 knots at 10%, 50% and 90% percentiles of the distribution, which were combined using multivariate meta-analysis [57, 58]. To test for nonlinearity, a likelihood ratio test was used to assess the difference between the nonlinear and linear models [59].

The Q test and I^2^ [60] were used to assess heterogeneity. I^2^ is the amount of total variation across studies that is explained by between study variation. I^2^ values of approximately 25%, 50% and 75% are considered to indicate low, moderate and high heterogeneity, respectively. Stratified analyses by study characteristics such as ethnicity, sex, duration of follow-up, geographic location, number of cases, study quality and adjustment for potential confounding and intermediate factors were conducted to investigate potential sources of heterogeneity. Publication bias was assessed with Egger’s test [61] and Begg’s test [62] and by inspection of funnel plots and the results were considered to indicate publication bias when *p* < 0.10 or if there was asymmetry in the funnel plots. We conducted sensitivity analyses excluding one study at a time to ensure that results were not simply due to one large study or a study with an extreme result. We also conducted an analysis stratified by whether conversions were made to obtain MET-hours/week or whether MET-hours/week was reported directly in the analysis of leisure-time physical activity. The statistical analyses were conducted using Stata, version 13.1 software (StataCorp, College Station, TX, USA).

## Results

**Fig. 1:**
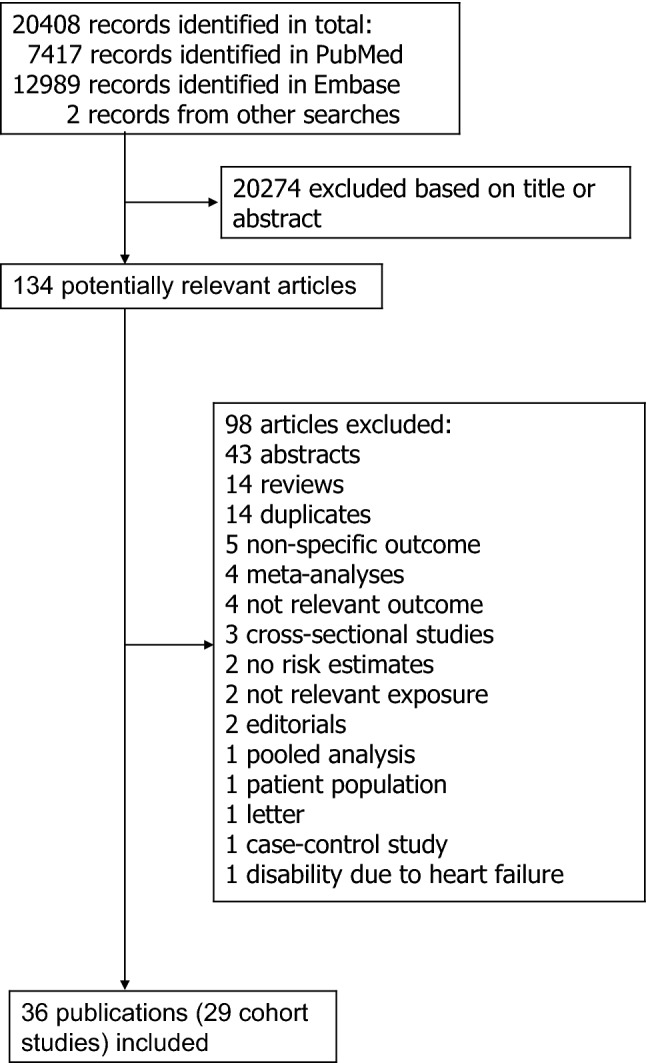
Flow-chart of study selection

Out of a total of 20,408 records identified by the search we included 29 prospective studies (36 publications) [12–47] in the systematic review of physical activity and cardiorespiratory fitness and risk of heart failure (Supplementary Tables 2, 3) and 27 of these studies (34 publications) [12–36, 38–46] were included in the meta-analyses. The meta-analysed studies included 21 prospective studies (25 publications) on physical activity including different domains of activity (Supplementary Table 2, Fig. 1) [12–35, 39, 40] and 6 prospective studies (7 publications) on cardiorespiratory fitness [35, 41–46] and risk of heart failure. Eleven studies on physical activity and heart failure were from the US, one from Canada, eight were from Europe, and one was an international study (Supplementary Table 2) while three studies on cardiorespiratory fitness and heart failure were from the U.S. and three were from Europe (Supplementary Table 2). Information on how cardiorespiratory fitness was assessed across studies is shown in Supplementary Table 4 and the definition of heart failure across studies is provided in Supplementary Table 5.

### Total physical activity

**Fig. 2:**
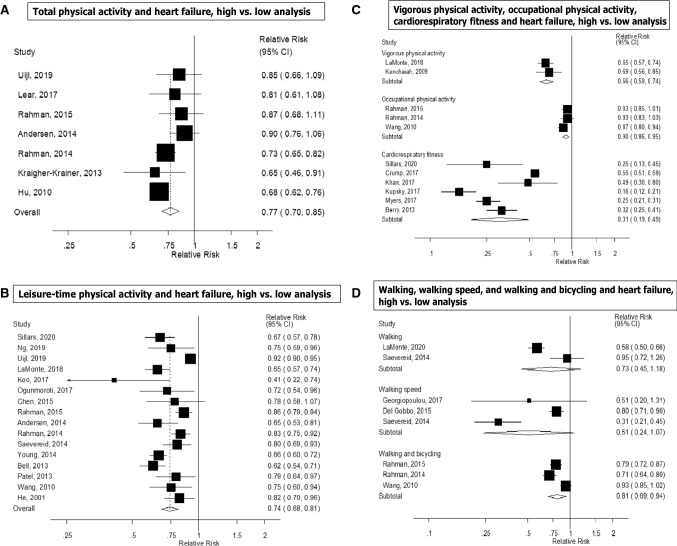
Total activity, leisure-time activity, vigorous activity, walking, walking speed, walking and bicycling combined, occupational activity, and cardiorespiratory fitness and heart failure, high versus low analysis

**Fig. 3:**
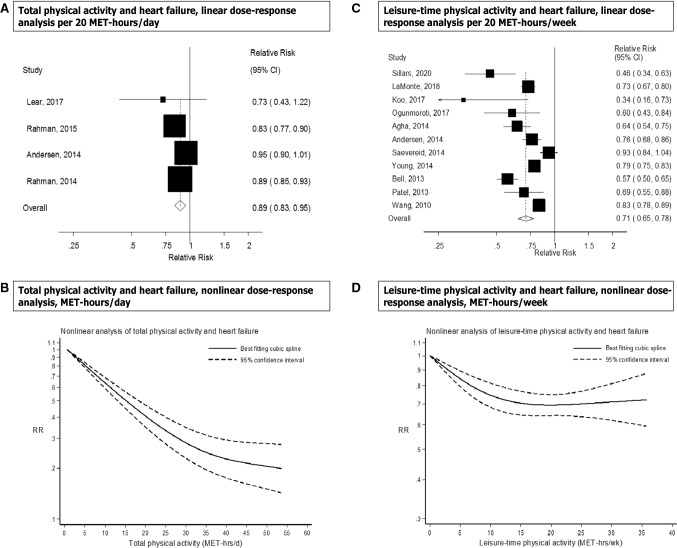
Total activity and leisure-time activity and heart failure, linear and nonlinear dose–response analyses

Seven prospective studies [14, 18, 21, 22, 26, 29, 32] were included in the high versus low analysis of total physical activity and heart failure risk, which included 12,496 cases and 329,768 participants. The summary RR for high versus low physical activity was 0.77 (95% CI 0.70–0.85, I^2^ = 48.7%, p_heterogeneity_ = 0.07) (Fig. 2a). There was no indication of publication bias with Egger’s test (*p* = 0.25) or with Begg’s test (*p* = 0.71) and there was no evidence of asymmetry by inspection of the funnel plot (Supplementary Figure 1). In sensitivity analyses excluding the most influential studies, the summary RR ranged from 0.74 (95% CI 0.68–0.80) when excluding the Sweden National March Study [21] to 0.80 (95% CI 0.73–0.88) when excluding the Finnish MONICA Study [14] (Supplementary Figure 2). Four prospective studies [21, 22, 26, 29] (7942 cases, 231,645 participants) were included in the dose–response analysis. The summary RR was 0.89 (95% CI 0.83–0.95, I^2^ = 66.8%, p_heterogeneity_ = 0.03, n = 4) per 20 MET-hours per day of total activity (Fig. 3a). Although the test for nonlinearity was significant (p_nonlinearity_ = 0.03) the association was approximately linear up to 25–30 MET-hours per day, and modest further reductions in risk were observed above that level of activity (Fig. 3b, Supplementary Table 6). In a sensitivity analysis we repeated the high versus low analysis with the same studies that were included in the dose–response meta-analysis and the summary RR was 0.81 (95% CI 0.72–0.91, I^2^ = 36.8%, p_heterogeneity_ = 0.19).

Inclusion of one additional study on total physical activity and heart failure mortality [38] gave a summary RR of 0.76 (95% CI 0.68–0.85, I^2^ = 55.6%, p_heterogeneity_ = 0.03) for high versus low activity (Supplementary Figure 3) (8125 cases, 290,767 participants) and 0.87 (95% CI 0.81–0.94, I^2^ = 71.5%, p_heterogeneity_ = 0.007) per 20 MET-hours/day (Supplementary Figure 6), and the results from the nonlinear meta-analysis also remained similar (Supplementary Table 6).

### Leisure-time physical activity

Sixteen prospective studies [12, 15–17, 19–22, 24, 26–28, 31, 33–35] were included in the analysis of leisure-time physical activity and risk of heart failure (> 73,391 cases and 1,895,300 participants). The summary RR for high versus low leisure-time activity was 0.74 (95% CI 0.68–0.81, I^2^ = 88.0%, p_heterogeneity_ < 0.0001) (Fig. 2b). There was evidence of publication bias with Egger’s test (*p* = 0.001) and by inspection of the funnel plot (Supplementary Figure 5), but not with Begg’s test (*p* = 0.75). However, this appeared to be driven by a large study [34] that only had a dichotomous categorization of physical activity (active vs. sedentary) and showed a weaker association than the remaining studies. When that study was excluded, there was no indication of publication bias with Egger’s test (*p* = 0.33) (Supplementary Figure 6), the summary estimate remained similar and there was less heterogeneity in the analysis (summary RR: 0.73, 95% CI 0.68–0.79, I^2^ = 65.5%, p_heterogeneity_ < 0.0001). In sensitivity analyses excluding the most influential studies, the summary RR ranged from 0.73 (95% CI 0.68–0.79) when excluding the Caliber study [34] to 0.75 (95% CI 0.69–0.82) when excluding the Atherosclerosis Risk in Communities Study [16] (Supplementary Figure 7). Eleven prospective studies [15–17, 19–21, 23, 27, 28, 31, 35] were included in the dose–response meta-analysis of leisure-time physical activity and risk of heart failure (19,582 cases and 919,498 participants) and the summary RR per 20 MET-hours per week was 0.71 (95% CI 0.65–0.78, I^2^ = 84.7%, p_heterogeneity_ < 0.0001) (Fig. 3c). There was indication of publication bias with Egger’s test (*p* = 0.04) and by inspection of the funnel plot (Supplementary Figure 8), and Begg’s test (*p* = 0.09), but this was driven by two outlying studies [27, 35], and when excluded, Egger’s test showed *p* = 0.17, and Begg’s test showed *p* = 0.25, and the results were similar, showing a summary RR of 0.74 (95% CI 0.68–0.81, I^2^ = 84.0%, p_heterogeneity_ < 0.0001). There was evidence of a nonlinear association, p_nonlinearity_ < 0.0001, with a reduction in risk observed up to between 15 and 20 MET-hours per week, but no further reductions in risk with higher levels of physical activity (Fig. 3d, Supplementary Table 6). In a sensitivity analysis we repeated the high versus low meta-analysis with the same studies included as in the dose–response meta-analysis and the summary RR was 0.68 (95% CI 0.65–0.72, I^2^ = 23%, p_heterogeneity_ = 0.22).

Inclusion of one additional study on heart failure mortality [40] did not alter the results (73,444 cases, 1,937,322 participants) and the summary RR was 0.74 (95% CI 0.68-0.81, I^2^ = 87.2%, p_heterogeneity_ < 0.0001) for high versus low leisure-time physical activity (Supplementary Figure 9) and 0.72 (95% CI 0.65–0.79, I^2^ = 83.3%, p_heterogeneity_ < 0.0001) per 20 MET-hours/week (Supplementary Figure 10). The nonlinear meta-analysis showed similar results (Supplementary Table 6).

**Fig. 4:**
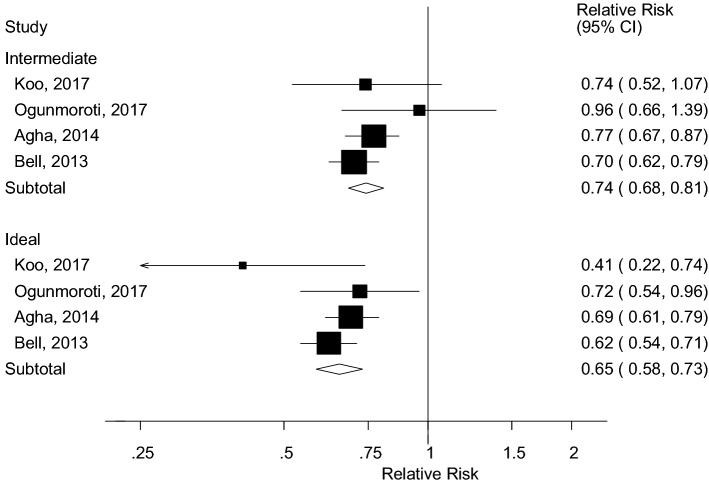
Leisure-time physical activity and heart failure, physical activity recommendations

Four studies (4004 cases, 108,834 participants) reported on leisure-time physical activity according to the physical activity recommendations [16, 23, 27, 28] in relation to heart failure and categorized physical activity as inactive (no activity), somewhat active or intermediate (< 150 min/week of moderate activity or < 75 min/week of vigorous activity), and active or ideal (≥ 150 min/week of moderate activity or ≥ 75 min/week of vigorous activity). The summary RRs were 0.74 (95% CI 0.68–0.81, I^2^ = 1.2%, p_heterogeneity_ = 0.39) for the intermediate category and 0.65 (0.58–0.73, I^2^ = 24.0%, p_heterogeneity_ = 0.27) for the ideal category compared to the inactive group, respectively (Fig. 4).

### Vigorous physical activity

**Fig. 5:**
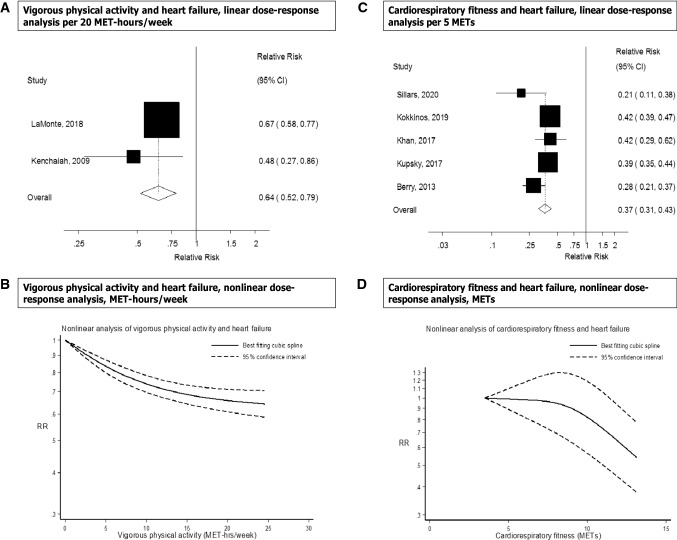
Vigorous physical activity and cardiorespiratory fitness and heart failure, linear and nonlinear dose–response analyses

Two prospective studies were included in the analysis of vigorous physical activity and risk of heart failure (3632 cases, 158,397 participants). The summary RR for high versus low vigorous physical activity was 0.66 (95% CI 0.59–0.74, I^2^ = 0%, p_heterogeneity_ = 0.63) (Fig. 2c) and per 20 MET-hours/week was 0.64 (95% CI 0.52–0.79, I^2^ = 14.9%, p_heterogeneity_ = 0.28) (Fig. 5a). There was some indication of a nonlinear association between vigorous activity and heart failure risk (p_nonlinearity_ < 0.0001), and the association was steeper up to 5–10 MET-hours/week of vigorous activity than at higher levels (Fig. 5b, Supplementary Table 6).

### Walking, walking speed and walking and bicycling combined

Two prospective studies [20, 40] were included in the analysis of walking and risk of heart failure (4103 cases and 155,512 participants). The summary RR for high versus low walking was 0.73 (95% CI 0.45–1.18, I^2^ = 89.6%, p_heterogeneity_ = 0.002) (Fig. 2d). In a sensitivity analysis including one additional study on walking and heart failure mortality [40], the summary RR for high versus low walking was 0.69 (95% CI 0.49–0.98, I^2^ = 79.4%, p_heterogeneity_ = 0.008) (4156 cases, 197,534 participants) (Supplementary Fig. 11).

Three prospective studies [20, 25, 30] were included in the meta-analysis of walking speed and risk of heart failure (3213 cases, 24,944 participants). The summary RR for high versus low walking speed was 0.51 (95% CI 0.24–1.07, I^2^ = 91.0%, p_heterogeneity_ < 0.0001) (Fig. 2d).

Three prospective studies [15, 22, 26] were included in the meta-analysis of walking and bicycling combined and risk of heart failure (9519 cases, 119,205 participants). The summary RR for high versus low walking and bicycling was 0.81 (95% CI 0.69–0.94, I^2^ = 86%, p_heterogeneity_ = 0.001) (Fig. 2d).

### Occupational physical activity

Three prospective studies [15, 22, 26] were included in the meta-analysis of occupational activity and risk of heart failure (9519 cases and 119,205 participants) and the summary RR for high versus low occupational activity was 0.90 (95% CI 0.86–0.95, I^2^ = 0%, p_heterogeneity_ = 0.46) (Fig. 2c).

### Cardiorespiratory fitness

Six studies [35, 41–45, 63] were included in the analysis of cardiorespiratory fitness and heart failure risk and included 19,693 cases and 1,505,114 participants. The summary RR for high versus low fitness was 0.31 (95% CI 0.19–0.49, I^2^ = 96.1%, p_heterogeneity_ < 0.0001) (Fig. 2c). There was no evidence of publication bias with Egger’s test (*p* = 0.11) or with Begg’s test (*p* = 0.85) and little indication of asymmetry in the funnel plot (Supplementary Figure 12). The summary RR ranged from 0.27 (95% CI 0.19–0.37) when the Swedish Military Conscription Database [43] was excluded to 0.36 (95% CI 0.23–0.55) when the Henry Ford Exercise Testing Project [44] was excluded (Supplementary Figure 13). Four studies (9059 cases, 173,678 participants) [35, 41, 44–46] were included in the linear dose–response meta-analysis of cardiorespiratory fitness and heart failure risk. The summary RR per 5 METs increase at exhaustion on the exercise test was 0.39 (95% CI 0.33–0.47, I^2^ = 70.0%, p_heterogeneity_ = 0.02) (Fig. 5c). There was evidence of a nonlinear association between cardiorespiratory fitness and heart failure (p_nonlinearity_ < 0.0001), with a threshold effect at around 12 METs and a reduced risk from that level of fitness and above (Fig. 5d, Supplementary Table 6).

### Subgroup and sensitivity analyses and study quality

The inverse associations between total physical activity, leisure-time physical activity, and cardiorespiratory fitness and risk of heart failure persisted in nearly all subgroup analyses defined by sex, duration of follow-up, geographic location, number of cases, study quality and adjustment for confounding factors including age, education, family history of cardiovascular disease, BMI, abdominal fatness, smoking, alcohol and potential intermediate factors such as hypertension, diabetes mellitus, triglycerides, cholesterol, history of coronary heart disease, interim coronary heart disease, valvular heart disease, left ventricular hypertrophy and medication use (ACE inhibitors, beta-blockers, diuretic drugs, antihypertensive medications, lipid-lowering medications, cardiovascular disease drugs), although there were few studies in some subgroups (Table 1). For total physical activity the association was stronger among studies that adjusted for alcohol consumption compared to those that did not (*p* = 0.04) (Table 1). For leisure-time activity there was indication of heterogeneity between subgroups when stratified by geographic location (*p* = 0.05) with a slightly stronger association observed in the American than in the European studies (Table 1). In analyses stratified by ethnicity, inverse associations were observed between high versus low leisure-time physical activity and risk of heart failure in Caucasians (summary RR = 0.66, 95% CI 0.61–0.72, I^2^ = 0%, p_heterogeneity_ = 0.50, n = 4), African Americans (summary RR = 0.67, 95% CI 0.57–0.78, I^2^ = 4%, p_heterogeneity_ = 0.37, n = 4), Hispanics (summary RR = 0.64, 95% CI 0.39–1.04, I^2^ = 58%, p_heterogeneity_ = 0.50, n = 3), and Asians (summary RR = 0.73, 95% CI 0.51–1.05, I^2^ = 5%, p_heterogeneity_ = 0.30, n = 2), although the associations were not statistically significant in the two latter subgroups (Supplementary Figure 14). However, there was no heterogeneity between these subgroups with meta-regression analyses (*p* = 0.99). In a sensitivity analysis, the summary RR per 20 MET-hours/week of leisure-time physical activity was 0.69 (95% CI 0.57–0.83, I^2^ = 90%, p_heterogeneity_ < 0.0001, n = 6) for studies where conversions were made in estimating MET-hours/week and 0.72 (95% CI 0.66–0.80, I^2^ = 71%, p_heterogeneity_ = 0.009, n = 5) for studies reporting on MET-hours/week directly (Supplementary Figure 15).

**Table 1:** Subgroup analyses of total physical activity, leisure-time physical activity and cardiorespiratory fitness and heart failure risk, high versus low analysis

	Total physical activity					Leisure-time physical activity					Cardiorespiratory fitness				
	*n*	RR (95% CI)	*I*^2^ (%)	*P*_h_^a^	*P*_h_^b^	*n*	RR (95% CI)	*I*^2^ (%)	*P*_h_^a^	*P*_h_^b^	*n*	RR (95% CI)	*I*^2^ (%)	*P*_h_^a^	*P*_h_^b^
All studies	7	0.77 (0.70–0.85)	48.7	0.07		16	0.74 (0.68–0.81)	88.0	< 0.0001		6	0.31 (0.19–0.49)	96.1	< 0.0001	
Duration of follow-up															
< 10 years follow-up	1	0.81 (0.62–1.08)			0.79	6	0.72 (0.59–0.88)	92.7	< 0.0001	0.85	3	0.23 (0.14–0.39)	84.9	< 0.0001	0.19
≥ 10 years follow-up	6	0.77 (0.69–0.86)	56.1	0.04		10	0.75 (0.69–0.82)	67.9	0.001		3	0.40 (0.22–0.74)	96.4	< 0.0001	
Sex															
Men	3	0.76 (0.66–0.87)	35.1	0.21	0.93/0.89^c^	9	0.79 (0.71–0.89)	87.1	< 0.0001	0.20/0.48^c^	4	0.42 (0.28–0.62)	83.3	< 0.0001	0.03/0.40^c^
Women	3	0.79 (0.64–0.99)	81.1	0.005		10	0.73 (0.61–0.86)	93.3	< 0.0001		2	0.31 (0.19–0.50)	2.5	0.31	
Men and women	3	0.79 (0.67–0.93)	0	0.45		5	0.69 (0.59–0.80)	45.4	0.12		2	0.20 (0.13–0.31)	84.8	0.01	
Geographic location															
Europe	5	0.78 (0.69–0.88)	62.8	0.03	0.99	7	0.80 (0.73–0.88)	82.0	< 0.0001	0.05	3	0.44 (0.30–0.67)	68.2	0.04	0.11
America	1	0.65 (0.46–0.91)				9	0.70 (0.64–0.76)	43.6	0.08		3	0.24 (0.16–0.34)	85.1	0.001	
International	1	0.81 (0.61–1.08)				0					0				
Number of cases															
Cases < 250	0				0.93	1	0.41 (0.22–0.74)			0.26	2	0.36 (0.19–0.69)	64.0	0.10	0.70
Cases 250– < 1000	3	0.79 (0.67–0.93)	0	0.45		2	0.75 (0.61–0.92)	0	0.71		0				
Cases ≥ 1000	4	0.77 (0.68–0.88)	69.0	0.02		12	0.75 (0.68–0.83)	90.5	< 0.0001		4	0.29 (0.16–0.52)	97.6	< 0.0001	
Study quality															
0–3	0				0.75	0				0.82	0				NC
4–6	1	0.81 (0.61–1.08)				1	0.78 (0.58–1.07)				0				
7–9	6	0.77 (0.69–0.86)	56.1	0.04		15	0.74 (0.67–0.81)	88.7	< 0.0001		6	0.32 (0.19–0.53)	96.8	< 0.0001	
*Adjustment for confounding factors*															
Age															
Yes	7	0.77 (0.70–0.85)	48.7	0.07	NC	16	0.74 (0.68–0.81)	88.0	< 0.0001	NC	6	0.31 (0.19–0.49)	96.1	< 0.0001	NC
No	0					0					0				
Education															
Yes	5	0.74 (0.68–0.82)	30.5	0.22	0.48	11	0.74 (0.68–0.81)	70.6	< 0.0001	0.82	1	0.55 (0.51–0.59)			0.15
No	2	0.79 (0.58–1.08)	64.6	0.09		5	0.73 (0.60–0.89)	87.8	< 0.0001		5	0.27 (0.19–0.37)	80.7	< 0.0001	
Family history of heart failure/CVD															
Yes	3	0.76 (0.69–0.84)	0	0.40	0.84	3	0.84 (0.79–0.89)	0	0.69	0.11	1	0.55 (0.51–0.59)			0.15
No	4	0.77 (0.65–0.91)	69.1	0.02		13	0.71 (0.63–0.81)	90.3	< 0.0001		5	0.27 (0.19–0.37)	80.7	< 0.0001	
Body mass index															
Yes	6	0.77 (0.69–0.86)	56.1	0.04	0.79	9	0.76 (0.69–0.83)	69.9	0.001	0.72	5	0.32 (0.19–0.53)	96.8	< 0.0001	0.71
No	1	0.81 (0.61–1.08)				7	0.73 (0.62–0.86)	91.8	< 0.0001		1	0.25 (0.13–0.45)			
Waist circumference, or waist-to-hip ratio															
Yes	2	0.80 (0.65–0.99)	75.5	0.04	0.63	2	0.75 (0.59–0.95)	75.9	0.04	0.92	0				NC
No	5	0.75 (0.66–0.85)	35.1	0.19		14	0.74 (0.67–0.82)	89.0	< 0.0001		6	0.31 (0.19–0.49)	96.1	< 0.0001	
Smoking															
Yes	6	0.78 (0.70–0.87)	54.6	0.05	0.41	11	0.73 (0.67–0.80)	75.4	< 0.0001	0.43	4	0.27 (0.19–0.39)	85.5	< 0.0001	0.41
No	1	0.65 (0.46–0.91)				5	0.77 (0.65–0.92)	81.1	< 0.0001		2	0.39 (0.18–0.85)	83.6	0.01	
Alcohol															
Yes	4	0.72 (0.66–0.78)	21.2	0.28	0.04	9	0.75 (0.68–0.82)	77.2	< 0.0001	0.91	1	0.49 (0.30–0.80)			0.39
No	3	0.87 (0.77–0.99)	0	0.81		7	0.72 (0.61–0.84)	86.7	< 0.0001		5	0.28 (0.17–0.48)	96.9	< 0.0001	
Hypertension															
Yes	4	0.79 (0.69–0.91)	49.5	0.11	0.59	10	0.77 (0.70–0.86)	87.0	< 0.0001	0.12	2	0.20 (0.13–0.31)	84.8	< 0.0001	0.08
No	3	0.74 (0.64–0.87)	42.6	0.18		6	0.69 (0.63–0.75)	22.2	0.26		4	0.40 (0.27–0.59)	86.7	< 0.0001	
Diabetes mellitus															
Yes	5	0.76 (0.67–0.85)	61.0	0.04	0.51	9	0.76 (0.69–0.83)	70.2	0.001	0.59	4	0.27 (0.19–0.39)	85.5	< 0.0001	0.41
No	2	0.83 (0.69–1.00)	0	0.80		7	0.72 (0.61–0.87)	91.7	< 0.0001		2	0.39 (0.18–0.85)	83.6	0.01	
Triglycerides															
Yes	0				NC	4	0.75 (0.65–0.87)	86.2	< 0.0001	0.86	0				NC
No	7	0.77 (0.70–0.85)	48.7	0.07		12	0.74 (0.65–0.83)	86.8	< 0.0001		6	0.31 (0.19–0.49)	96.1	< 0.0001	
Serum cholesterol															
Yes	2	0.74 (0.60–0.91)	61.7	0.11	0.44	4	0.78 (0.63–0.96)	94.1	< 0.0001	0.45	1	0.32 (0.25–0.41)			0.95
No	5	0.79 (0.71–0.89)	33.3	0.20		12	0.73 (0.67–0.80)	68.3	< 0.0001		5	0.31 (0.17–0.54)	96.6	< 0.0001	
Prevalent coronary heart disease															
Yes	3	0.73 (0.65–0.81)	43.2	0.17	0.13	4	0.82 (0.73–0.91)	50.1	0.11	0.29	2	0.27 (0.09–0.82)	93.4	< 0.0001	0.66
No	4	0.84 (0.75–0.94)	0	0.41		12	0.73 (0.65–0.82)	90.7	< 0.0001		4	0.33 (0.20–0.54)	95.8	< 0.0001	
Interim coronary heart disease															
Yes	0				NC	1	0.82 (0.70–0.96)			0.51	1	0.55 (0.51–0.59)			0.15
No	7	0.77 (0.70–0.85)	48.7	0.07		15	0.74 (0.67–0.81)	88.8	< 0.0001		5	0.27 (0.19–0.37)	80.7	< 0.0001	
Valvular heart disease															
Yes	2	0.68 (0.61–0.75)	0	0.80	0.09	2	0.80 (0.70–0.91)	0	0.52	0.59	1	0.55 (0.51–0.59)			0.15
No	5	0.81 (0.74–0.89)	19.9	0.29		14	0.73 (0.66–0.81)	89.4	< 0.0001		5	0.27 (0.19–0.37)	80.7	< 0.0001	
Left ventricular hypertrophy															
Yes	1	0.65 (0.46–0.91)			0.41	1	0.79 (0.64–0.97)			0.72	0				0.57
No	6	0.78 (0.70–0.87)	54.6	0.05		15	0.74 (0.67–0.81)	88.7	< 0.0001		6	0.31 (0.19–0.49)	96.1	< 0.0001	
ACE inhibitor use															
Yes	0				NC	0				NC	2	0.20 (0.13–0.31)	84.8	0.01	0.08
No	7	0.77 (0.70–0.85)	48.7	0.07		16	0.74 (0.68–0.81)	88.0	< 0.0001		4	0.40 (0.27–0.59)	86.7	< 0.0001	
Beta-blocker use															
Yes	0				NC	0				NC	2	0.20 (0.13–0.31)	84.8	0.01	0.08
No	7	0.77 (0.70–0.85)	48.7	0.07		16	0.74 (0.68–0.81)	88.0	< 0.0001		4	0.40 (0.27–0.59)	86.7	< 0.0001	
Diuretic use															
Yes	0				NC	0				NC	2	0.20 (0.13–0.31)	84.8	0.01	0.08
No	7	0.77 (0.70–0.85)	48.7	0.07		16	0.74 (0.68–0.81)	88.0	< 0.0001		4	0.40 (0.27–0.59)	86.7	< 0.0001	
Antihypertensive medication use															
Yes	1	0.65 (0.46–0.91)			0.41	3	0.77 (0.60–1.00)	95.9	< 0.0001	0.54	2	0.20 (0.13–0.31)	84.8	0.01	0.08
No	6	0.78 (0.70–0.87)	54.6	0.05		13	0.74 (0.68–0.80)	65.6	< 0.0001		4	0.40 (0.27–0.59)	86.7	< 0.0001	
Lipid-lowering medication use															
Yes	0				NC	1	0.92 (0.90–0.95)			0.05	2	0.20 (0.13–0.31)	84.8	0.01	0.08
No	7	0.77 (0.70–0.85)	48.7	0.07		15	0.73 (0.68–0.79)	67.0	< 0.0001		4	0.40 (0.27–0.59)	86.7	< 0.0001	
CVD medication use															
Yes	0				NC	1	0.67 (0.57–0.78)			0.45	2	0.25 (0.21–0.30)	0	0.99	0.52
No	7	0.77 (0.70–0.85)	48.7	0.07		15	0.75 (0.68–0.82)	87.8	< 0.0001		4	0.34 (0.19–0.62)	96.4	< 0.0001	

*NC* not calculable

*n* number of studies

^a^P for heterogeneity within each subgroup

^b^P for heterogeneity between subgroups with meta-regression analysis

^c^P for heterogeneity between men and women (studies with genders combined were excluded)

The mean (median) study quality scores were 7.4 (8.0) in studies of total physical activity (Supplementary Table 5), 7.4 (7.0) in studies of leisure-time physical activity (Supplementary Table 6), and 7.7 (7.5) in studies of cardiorespiratory fitness (Supplementary Table 7).

## Discussion

In this comprehensive meta-analysis, high versus low levels of total physical activity, leisure-time activity, vigorous activity, walking and bicycling combined, occupational activity and cardiorespiratory fitness were each associated with a statistically significant decrease in the risk of heart failure. Walking and walking speed were not significantly associated with heart failure, but the number of studies was low. For total physical activity, leisure-time activity, and vigorous activity the inverse associations were most pronounced at lower levels of activity, while for cardiorespiratory fitness a threshold effect was observed from around 12 METs at the exercise test. Increasing compliance with the recommendations for leisure-time activity was also associated with a reduced risk of heart failure. The inverse association between leisure-time activity and heart failure was consistent across ethnic groups. Our findings are largely consistent with those of two previous meta-analyses [9, 10], however, one of these did not conduct dose–response meta-analyses [10] and neither of them investigated specific domains of physical activity or potential effect modification by ethnicity.

Although much is unknown regarding the biologic mechanisms that could explain the observed inverse association between physical activity and heart failure, both indirect and direct effects may contribute. Physical activity could reduce the risk of heart failure indirectly by improving body weight control and lowering risk of overweight and obesity and weight gain [64–66], improving insulin sensitivity [67] and lowering the risk of type 2 diabetes [56], reducing blood pressure and the risk of hypertension [66, 68–70], and lowering resting heart rate [66] and reducing the risk of coronary heart disease [71], as all these risk factors are associated with increased risk of heart failure [5, 6, 72]. However, in the current meta-analysis, there was little difference in the results between subgroups of studies that adjusted for BMI, diabetes and hypertension and those that did not. Also, two previous studies that made adjustments for BMI in a separate step within the same datasets found little difference in the results [13, 18]. This suggests that most of the association is independent of adiposity.

In addition to indirect effects, physical activity may also reduce the risk of heart failure directly by increasing myocardial oxygen supply, improving cardiac function, reducing interstitial fibrosis, and increasing capillary density [73, 74]. One study found that physical activity was associated with reduced risk of developing elevated levels of biomarkers of cardiac injury and hemodynamic stress including NT-proBNP and cTnT [75]. Physical activity has also been associated with reduced left ventricular mass and reduced risk of left ventricular hypertrophy in hypertensive and obese subjects [76, 77]. Although some studies found that healthy adults and highly trained athletes who were physically active also had greater left ventricular mass and hypertrophy [78, 79], it has been suggested that cardiac remodeling resulting from exercise is not pathologic because it lacks the fibrosis component seen in hypertension [80].

Our meta-analysis has some limitations that need to be mentioned. Confounding by other risk factors may have influenced the results. However, the association between physical activity or cardiorespiratory fitness and heart failure persisted in subgroup analyses defined by adjustments for confounding factors such as age, education, family history of cardiovascular disease, BMI, waist circumference, smoking, alcohol, as well as adjustments for potential intermediate factors such as hypertension, diabetes mellitus, triglycerides, serum cholesterol, and history of coronary heart disease, interim coronary heart disease, valvular heart disease, left ventricular hypertrophy. Although few studies adjusted for use of various medications, those that did were in general consistent with the overall findings. In meta-regression analyses, there was in general little evidence of heterogeneity between subgroups and in the few cases where heterogeneity was present, chance cannot be ruled out as a potential explanation. In the meta-analysis of leisure-time physical activity, there was some evidence of publication bias, however, this appeared to be driven largely by one outlying study and exclusion of that study did not materially alter the results. There was no evidence of publication bias in the meta-analysis of total physical activity or cardiorespiratory fitness. Accurate measurement of physical activity is a challenge and none of the included studies corrected for measurement errors. However, given the prospective design of the included studies, such measurement errors would most likely have led to an attenuation of the observed associations and an underestimation of the magnitude of the true RR. In addition, changes in physical activity levels over time could have influenced the results, but few of the included studies had repeated measures of physical activity during follow-up. Relatively few studies investigated specific types and intensities of physical activity and therefore further studies are needed on these exposures. Because not all studies reported the results in MET-hours/week we converted the quantities to MET-hours/week where this was possible. This could have impacted the summary estimates, however, in stratified analyses there was very little difference in the observed associations by whether conversions were made or whether studies reported on MET-hours/week directly. Lastly, as in our previous meta-analyses on physical activity and various health outcomes [56, 81, 82], we were not able to include all relevant studies in the dose–response meta-analyses because of a lack of information on the amount of physical activity or fitness in several studies. In sensitivity analyses restricting the high versus low analysis to the studies included in the dose–response analysis we found that this may have slightly exaggerated the results for leisure-time physical activity and slightly underestimated the association for total physical activity, however, these differences were relatively modest. It is important that future studies quantify the amount of physical activity either in minutes or hours per week or MET-minutes or MET-hours per week so that the results can be combined with the existing studies.

Strengths of our meta-analysis include the prospective design of the included studies, which avoided recall bias and reduced the possibility of selection bias. Also, the large sample size with up to 73,000 cases and ~ 1.9 million participants provided sufficient statistical power to detect even modest associations. Moreover, the nonlinear dose–response meta-analyses clarified the shape of the dose–response relationships. Additional merits include the robustness of the findings in multiple subgroup and sensitivity analyses, the high study quality scores of the included studies and the detailed analyses of specific domains of physical activity. The current findings have important public health implications as the incidence of heart failure is expected to increase with an ageing population [83]. Promotion of physical activity could therefore contribute towards primary prevention of heart failure in the general population, and our findings suggests a range of activities may have benefit including less intense activities, such as walking and bicycling, which may be easier to adhere to for elderly people that may be at risk of heart failure.

In conclusion, these findings suggest that higher levels of total physical activity, leisure-time activity, vigorous activity, walking and bicycling combined, occupational activity and cardiorespiratory fitness reduce the risk of developing heart failure. Our results support recommendations to increase the level of physical activity in the general population. Future studies should investigate the associations between specific domains of physical activity and subtypes of heart failure and report the results in a manner that can be included in dose–response meta-analyses. Further investigations of the underlying mechanisms are also warranted.

## Electronic supplementary material

Below is the link to the electronic supplementary material.

Supplementary material 1 (PDF 426 kb)

## References

[1] GBD 2015 Mortality and Causes of Death Collaborators (2016). Global, regional, and national life expectancy, all-cause mortality, and cause-specific mortality for 249 causes of death, 1980–2015: a systematic analysis for the Global Burden of Disease Study 2015. Lancet.

[2] Massie BM, Shah NB (1997). Evolving trends in the epidemiologic factors of heart failure: rationale for preventive strategies and comprehensive disease management. Am Heart J.

[3] Shahar E, Lee S, Kim J, Duval S, Barber C, Luepker RV (2004). Hospitalized heart failure: rates and long-term mortality. J Card Fail.

[4] Spencer FA, Meyer TE, Goldberg RJ (1999). Twenty year trends (1975–1995) in the incidence, in-hospital and long-term death rates associated with heart failure complicating acute myocardial infarction: a community-wide perspective. J Am Coll Cardiol.

[5] Aune D, Sen A, Norat T (2016). Body mass index, abdominal fatness and heart failure incidence and mortality: a systematic review and dose–response meta-analysis of prospective studies. Circulation.

[6] Aune D, Sen A, O’Hartaigh B (2017). Resting heart rate and the risk of cardiovascular disease, total cancer, and all-cause mortality—a systematic review and dose-response meta-analysis of prospective studies. Nutr Metab Cardiovasc Dis.

[7] Wilhelmsen L, Rosengren A, Eriksson H, Lappas G (2001). Heart failure in the general population of men–morbidity, risk factors and prognosis. J Intern Med.

[8] Ho JE, Lyass A, Lee DS (2013). Predictors of new-onset heart failure: differences in preserved versus reduced ejection fraction. Circ Heart Fail.

[9] Pandey A, Garg S, Khunger M (2015). Dose-response relationship between physical activity and risk of heart failure: a meta-analysis. Circulation.

[10] Echouffo-Tcheugui JB, Butler J, Yancy CW, Fonarow GC (2015). Association of physical activity or fitness with incident heart failure: a systematic review and meta-analysis. Circ Heart Fail.

[11] Kyu HH, Bachman VF, Alexander LT (2016). Physical activity and risk of breast cancer, colon cancer, diabetes, ischemic heart disease, and ischemic stroke events: systematic review and dose-response meta-analysis for the Global Burden of Disease Study 2013. BMJ.

[12] He J, Ogden LG, Bazzano LA, Vupputuri S, Loria C, Whelton PK (2001). Risk factors for congestive heart failure in US men and women: NHANES I epidemiologic follow-up study. Arch Intern Med.

[13] Kenchaiah S, Sesso HD, Gaziano JM (2009). Body mass index and vigorous physical activity and the risk of heart failure among men. Circulation.

[14] Hu G, Jousilahti P, Antikainen R, Katzmarzyk PT, Tuomilehto J (2010). Joint effects of physical activity, body mass index, waist circumference, and waist-to-hip ratio on the risk of heart failure. Circulation.

[15] Wang Y, Tuomilehto J, Jousilahti P (2010). Occupational, commuting, and leisure-time physical activity in relation to heart failure among finnish men and women. J Am Coll Cardiol.

[16] Bell EJ, Lutsey PL, Windham BG, Folsom AR (2013). Physical activity and cardiovascular disease in African Americans in atherosclerosis risk in communities. Med Sci Sports Exerc.

[17] Patel K, Sui X, Zhang Y (2013). Prevention of heart failure in older adults may require higher levels of physical activity than needed for other cardiovascular events. Int J Cardiol.

[18] Kraigher-Krainer E, Lyass A, Massaro JM (2013). Association of physical activity and heart failure with preserved versus reduced ejection fraction in the elderly: the Framingham Heart Study. Eur J Heart Fail.

[19] Young DR, Reynolds K, Sidell M (2014). Effects of physical activity and sedentary time on the risk of heart failure. Circ Heart Fail.

[20] Saevereid HA, Schnohr P, Prescott E (2014). Speed and duration of walking and other leisure time physical activity and the risk of heart failure: a prospective cohort study from the Copenhagen City Heart Study. PLoS ONE.

[21] Andersen K, Mariosa D, Adami HO (2014). Dose-response relationship of total and leisure time physical activity to risk of heart failure: a prospective cohort study. Circ Heart Fail.

[22] Rahman I, Bellavia A, Wolk A (2014). Relationship between physical activity and heart failure risk in women. Circ Heart Fail.

[23] Agha G, Loucks EB, Tinker LF (2014). Healthy lifestyle and decreasing risk of heart failure in women: the Women’s Health Initiative observational study. J Am Coll Cardiol.

[24] Chen Y, Sloan FA, Yashkin AP (2015). Adherence to diabetes guidelines for screening, physical activity and medication and onset of complications and death. J Diabetes Complicat.

[25] Del Gobbo LC, Kalantarian S, Imamura F (2015). Contribution of major lifestyle risk factors for incident heart failure in older adults: the Cardiovascular Health Study. JACC Heart Fail.

[26] Rahman I, Bellavia A, Wolk A, Orsini N (2015). Physical activity and heart failure risk in a prospective study of men. JACC Heart Fail.

[27] Koo P, Gjelsvik A, Choudhary G (2017). Prospective association of physical activity and heart failure hospitalizations among black adults with normal ejection fraction: the Jackson Heart Study. J Am Heart Assoc.

[28] Ogunmoroti O, Oni E, Michos ED (2017). Life’s simple 7 and incident heart failure: The Multi-Ethnic Study of atherosclerosis. J Am Heart Assoc.

[29] Lear SA, Hu W, Rangarajan S (2017). The effect of physical activity on mortality and cardiovascular disease in 130 000 people from 17 high-income, middle-income, and low-income countries: the PURE study. Lancet.

[30] Georgiopoulou VV, Kalogeropoulos AP, Chowdhury R (2017). Exercise capacity, heart failure risk, and mortality in older adults: The Health ABC Study. Am J Prev Med.

[31] LaMonte MJ, Manson JE, Chomistek AK (2018). Physical activity and incidence of heart failure in postmenopausal women. JACC Heart Fail.

[32] Uijl A, Koudstaal S, Vaartjes I (2019). Risk for Heart failure: the opportunity for prevention with the American Heart Association’s Life’s Simple 7. JACC Heart Fail.

[33] Ng R, Sutradhar R, Yao Z, Wodchis WP, Rosella LC. Smoking, drinking, diet and physical activity-modifiable lifestyle risk factors and their associations with age to first chronic disease. Int J Epidemiol. 2020;49(1):113–30. 10.1093/ije/dyz078 PMC7124486 31329872

[34] Uijl A, Koudstaal S, Direk K (2019). Risk factors for incident heart failure in age- and sex-specific strata: a population-based cohort using linked electronic health records. Eur J Heart Fail.

[35] Sillars A, Ho FK, Pell GP (2020). Sex differences in the association of risk factors for heart failure incidence and mortality. Heart.

[36] Williams PT, Thompson PD (2013). The relationship of walking intensity to total and cause-specific mortality. Results from the National Walkers’ Health Study. PLoS One.

[37] Florido R, Kwak L, Lazo M (2018). Six-year changes in physical activity and the risk of incident heart failure: ARIC study. Circulation.

[38] Hamer M, O’Donovan G, Stamatakis E. Association between physical activity and sub-types of cardiovascular disease death causes in a general population cohort. Eur J Epidemiol. 2019;34(5):483–7. 10.1007/s10654-018-0460-2 PMC6456476 30417220

[39] Eaton CB, Pettinger M, Rossouw J (2016). Risk factors for incident hospitalized heart failure with preserved versus reduced ejection fraction in a multiracial cohort of postmenopausal women. Circ Heart Fail.

[40] Williams PT (2013). Dose-response relationship of physical activity to premature and total all-cause and cardiovascular disease mortality in walkers. PLoS ONE.

[41] Berry JD, Pandey A, Gao A (2013). Physical fitness and risk for heart failure and coronary artery disease. Circ Heart Fail.

[42] Myers J, Kokkinos P, Chan K (2017). Cardiorespiratory fitness and reclassification of risk for incidence of heart failure: The Veterans Exercise Testing Study. Circ Heart Fail.

[43] Crump C, Sundquist J, Winkleby MA, Sundquist K (2017). Aerobic fitness, muscular strength and obesity in relation to risk of heart failure. Heart.

[44] Kupsky DF, Ahmed AM, Sakr S (2017). Cardiorespiratory fitness and incident heart failure: The Henry Ford ExercIse Testing (FIT) Project. Am Heart J.

[45] Khan H, Jaffar N, Rauramaa R, Kurl S, Savonen K, Laukkanen JA (2017). Cardiorespiratory fitness and nonfatalcardiovascular events: a population-based follow-up study. Am Heart J.

[46] Kokkinos P, Faselis C, Franklin B (2019). Cardiorespiratory fitness, body mass index and heart failure incidence. Eur J Heart Fail.

[47] Pandey A, Patel M, Gao A (2015). Changes in mid-life fitness predicts heart failure risk at a later age independent of interval development of cardiac and noncardiac risk factors: the Cooper Center Longitudinal Study. Am Heart J.

[48] GBD 2017 Risk Factor Collaborators (2018). Global, regional, and national comparative risk assessment of 84 behavioural, environmental and occupational, and metabolic risks or clusters of risks for 195 countries and territories, 1990–2017: a systematic analysis for the Global Burden of Disease Study 2017. Lancet.

[49] Moher D, Liberati A, Tetzlaff J, Altman DG (2009). Preferred reporting items for systematic reviews and meta-analyses: the PRISMA statement. BMJ.

[50] Wells G, Shea B, O’Connell D. et al. The Newcastle-Ottawa Scale (NOS) for assessing the quality of nonrandomised studies in meta-analyses. http://www.ohri.ca/programs/clinical_epidemiology/oxford.asp. Accessed 09 Aug 2018.

[51] Hsu CC, Fu TC, Yuan SS (2019). High-intensity interval training is associated with improved long-term survival in heart failure patients. J Clin Med.

[52] DerSimonian R, Laird N (1986). Meta-analysis in clinical trials. Control Clin Trials.

[53] Hamling J, Lee P, Weitkunat R, Ambuhl M (2008). Facilitating meta-analyses by deriving relative effect and precision estimates for alternative comparisons from a set of estimates presented by exposure level or disease category. Stat Med.

[54] Greenland S, Longnecker MP (1992). Methods for trend estimation from summarized dose-response data, with applications to meta-analysis. Am J Epidemiol.

[55] Aune D, Greenwood DC, Chan DS (2012). Body mass index, abdominal fatness and pancreatic cancer risk: a systematic review and non-linear dose-response meta-analysis of prospective studies. Ann Oncol.

[56] Aune D, Norat T, Leitzmann M, Tonstad S, Vatten LJ (2015). Physical activity and the risk of type 2 diabetes: a systematic review and dose-response meta-analysis. Eur J Epidemiol.

[57] Jackson D, White IR, Thompson SG (2010). Extending DerSimonian and Laird’s methodology to perform multivariate random effects meta-analyses. Stat Med.

[58] Orsini N, Li R, Wolk A, Khudyakov P, Spiegelman D (2012). Meta-analysis for linear and nonlinear dose-response relations: examples, an evaluation of approximations, and software. Am J Epidemiol.

[59] Royston P (2000). A strategy for modelling the effect of a continuous covariate in medicine and epidemiology. Stat Med.

[60] Higgins JP, Thompson SG (2002). Quantifying heterogeneity in a meta-analysis. Stat Med.

[61] Egger M, Davey SG, Schneider M, Minder C (1997). Bias in meta-analysis detected by a simple, graphical test. BMJ.

[62] Begg CB, Mazumdar M (1994). Operating characteristics of a rank correlation test for publication bias. Biometrics.

[63] Khan H, Kunutsor S, Rauramaa R (2014). Cardiorespiratory fitness and risk of heart failure: a population-based follow-up study. Eur J Heart Fail.

[64] Mozaffarian D, Hao T, Rimm EB, Willett WC, Hu FB (2011). Changes in diet and lifestyle and long-term weight gain in women and men. N Engl J Med.

[65] Ekelund U, Besson H, Luan J (2011). Physical activity and gain in abdominal adiposity and body weight: prospective cohort study in 288,498 men and women. Am J Clin Nutr.

[66] Hespanhol Junior LC, Pillay JD, van MW, Verhagen E (2015). Meta-analyses of the effects of habitual running on indices of health in physically inactive adults. Sports Med.

[67] Mayer-Davis EJ, D’Agostino R, Karter AJ (1998). Intensity and amount of physical activity in relation to insulin sensitivity: the Insulin Resistance Atherosclerosis Study. JAMA.

[68] Hollingworth M, Harper A, Hamer M (2015). Dose-response associations between cycling activity and risk of hypertension in regular cyclists: The UK Cycling for Health Study. J Hum Hypertens.

[69] Fagard RH (2001). Exercise characteristics and the blood pressure response to dynamic physical training. Med Sci Sports Exerc.

[70] Figueira FR, Umpierre D, Cureau FV (2014). Association between physical activity advice only or structured exercise training with blood pressure levels in patients with type 2 diabetes: a systematic review and meta-analysis. Sports Med.

[71] Sofi F, Capalbo A, Cesari F, Abbate R, Gensini GF (2008). Physical activity during leisure time and primary prevention of coronary heart disease: an updated meta-analysis of cohort studies. Eur J Cardiovasc Prev Rehabil.

[72] Johansson S, Wallander MA, Ruigomez A, Garcia Rodriguez LA (2001). Incidence of newly diagnosed heart failure in UK general practice. Eur J Heart Fail.

[73] Miyachi M, Yazawa H, Furukawa M (2009). Exercise training alters left ventricular geometry and attenuates heart failure in dahl salt-sensitive hypertensive rats. Hypertension.

[74] Emter CA, Tharp DL, Ivey JR, Ganjam VK, Bowles DK (2011). Low-intensity interval exercise training attenuates coronary vascular dysfunction and preserves Ca(2)(+)-sensitive K(+) current in miniature swine with LV hypertrophy. Am J Physiol Heart Circ Physiol.

[75] deFilippi CR, de Lemos JA, Tkaczuk AT (2012). Physical activity, change in biomarkers of myocardial stress and injury, and subsequent heart failure risk in older adults. J Am Coll Cardiol.

[76] Kamimura D, Loprinzi PD, Wang W (2017). Physical activity is associated with reduced left ventricular mass in obese and hypertensive African Americans. Am J Hypertens.

[77] Palatini P, Visentin P, Dorigatti F (2009). Regular physical activity prevents development of left ventricular hypertrophy in hypertension. Eur Heart J.

[78] Maskhulia L, Chabashvili N, Akhalkatsi V, Chutkerashvili T. Left ventricular morphological changes due to vigorous physical activity in highly trained football players and wrestlers: relationship with aerobic capacity. Georgian Med News 2006;133:68–71. 16705233

[79] Dawes TJ, Corden B, Cotter S (2016). Moderate physical activity in healthy adults is associated with cardiac remodeling. Circ Cardiovasc Imaging.

[80] Hegde SM, Solomon SD (2015). Influence of physical activity on hypertension and cardiac structure and function. Curr Hypertens Rep.

[81] Aune D, Sen A, Henriksen T, Saugstad OD, Tonstad S (2016). Physical activity and the risk of gestational diabetes mellitus: a systematic review and dose-response meta-analysis of epidemiological studies. Eur J Epidemiol.

[82] Aune D, Leitzmann M, Vatten LJ (2016). Physical activity and the risk of gallbladder disease: a systematic review and meta-analysis of cohort studies. J Phys Act Health.

[83] Savarese G, Lund LH (2017). Global public health burden of heart failure. Card Fail Rev.

