# Supplementary material for: Physical activity and the risk of heart failure: a systematic review and dose–response meta-analysis of prospective studies
Source: Eur J Epidemiol. 2020 Dec 17;36(4):367–81. doi: 10.1007/s10654-020-00693-6 (PMC8076120; doi:10.1007/s10654-020-00693-6)

Supplementary Text

Search strategy in PubMed

(“physical activity” OR exercise OR sports OR walking OR biking OR running OR fitness OR “exercise test” OR inactivity OR sedentary)

AND

"heart failure"[MeSH Terms] OR "heart failure"

AND

"case-control" OR retrospective OR cohort OR cohorts OR prospective OR longitudinal OR "follow-up" OR "cross-sectional" OR trial OR "relative risk" OR "risk ratio" OR "hazard ratio" OR "odds ratio"

Search strategy in Embase

((physical activity or exercise or sports or walking or biking or running or fitness or exercise test or inactivity or sedentary).ti,ab.

OR

physical activity/ or cycling/ or jogging/ or running/ or walking/ or bicycle/ or sport/ or exercise/ or fitness/ or sedentary lifestyle/)

AND

(exp heart failure/co, ep, et, pc [Complication, Epidemiology, Etiology, Prevention]

OR

heart failure/

OR

heart failure.ab,ti.)

AND

(case-control or retrospective or cohort or cohorts or prospective or longitudinal or follow-up or cross-sectional or trial or relative risk or risk ratio or hazard ratio or odds ratio).af.

Supplementary Table 1. List of studies excluded and exclusion reason

Exclusion reason	Reference number
Abstract	(1-43)
Case-control study	(44)
Cross-sectional study	(45-47)
Disability due to heart failure	(48)
Duplicate	(49-62)
Editorial, letter	(63-65)
Meta-analysis	(66-69)
No risk estimates	(70;71)
Not relevant exposure	(72;73)
Not relevant outcome	(74-77)
Patient population	(78)
Pooled analysis	(79)
Review	(80-93)
Unspecific outcome	(94-98)

Reference List

1. Singh S, Zhang Y, Desai R et al. Physical activity and incident heart failure in community dwelling older adults with and without metabolic syndrome. Circulation Conference: American Heart Association's Scientific Sessions 2011;22.

2. Turkbey EB, Jorgensen NW, Bertoni AG et al. Physical activity and cardiovascular events in the multi-ethnic study of atherosclerosis (MESA). Circulation Conference: American Heart Association's Scientific Sessions 2011;22.

3. Pandey A, Willis BL, Gao A et al. Midlife cardiorespiratory fitness predicts late-life risk for heart failure independent of interval development of hypertension, diabetes, and myocardial infarction. Circulation Conference: American Heart Association 2012;20.

4. Bell EJ. Strong inverse relation between physical activity and incidence of cardiovascular disease in african americans: The atherosclerosis risk in communities study. Circulation Conference: Epidemiology and Prevention/Physical Activity, Nutrition and Metabolism 2012;13.

5. Andersen K, Mariosa D, Adami HO et al. Total and leisure time physical activity and risk of heart failure: A prospective cohort study. European Heart Journal Conference: ESC Congress 2012;August.

6. Andersen K, Mariosa D, Adami H-O et al. Total and leisure time physical activity and risk of heart failure: A prospective cohort study. Scandinavian Cardiovascular Journal Conference: 14th Svenska Kardiovaskulara Varmotet Stockholm Sweden Conference Publication: 2012;46 (SUPPL 60);24.

7. Saevereid HA, Peter Schnohr PS, Eva Irene Bossano Prescott EP. Volume and intensity of exercise and the risk of heart failure: the Copenhagen City Heart Study. European Journal of Preventive Cardiology Conference: EuroPrevent 2013;April.

8. Cornwell WK, Neeland I, Pandey A et al. Combined association of midlife obesity and fitness with longterm risk of heart failure-the cooper center longitudinal study. Circulation Conference: American Heart Association 2013;26.

9. Khan H, Kunutsor S, Rauramaa R et al. Cardio-respiratory fitness and incident heart failure. Circulation Conference: American Heart Association 2013;26.

10. Young DR, Reynolds K, Sidell MA et al. Effects of physical activity and sedentary behaviors on risk of heart failure. Circulation Conference: American Heart Association's Epidemiology and Prevention/Physical Activity, Nutrition and Metabolism 2013;26.

11. Agha G, Loucks EB, Tinker L et al. Healthy lifestyle factors and risk of heart failure in the women's health initiative observational study. Circulation Conference: American Heart Association's Epidemiology and Prevention/Physical Activity, Nutrition and Metabolism 2013;26.

12. Saevereid HAS, Schnohr PS, Prescott EP. Speed and duration of walking and other leisure time physical activity and the risk of heart failure: The Copenhagen City Heart study. European Heart Journal Conference: European Society of Cardiology, ESC Congress 2013;August.

13. Pandey A, Willis B, Leonard D, DeFina L, Gao A, Berry J. Change in midlife fitness is an independent predictor of heart failure in older age. Circulation: Cardiovascular Quality and Outcomes Conference: American Heart Association's Quality of Care and Outcomes Research in Cardiovascular Disease and Stroke 2013;May.

14. Engeseth K, Skretteberg PT, Grundvold I et al. Physical fitness predicts myocardial infarction and heart failure but only when age-adjusted heart rate response to maximal exercise is low. European Heart Journal Conference: European Society of Cardiology, ESC Congress 2014;01.

15. Del GL, Kalantarian S, Imamura F, Lemaitre R, Siscovick D, Mozaffarian D. Contribution of preventable risk factors for incident congestive heart failure in older adults: The cardiovascular health study. Circulation Conference: American Heart Association's Epidemiology and Prevention/Nutrition, Physical Activity, and Metabolism 2014;25.

16. Khan H, Rauramaa R, Kurl S, Savonen K, Laukkanen JA. Cardiorespiratory fitness and non fatal cardiovascular events. Circulation Conference: American Heart Association's 2015;10.

17. Florido R, Kwak L, Lazo M et al. Changes in physical activity and the risk of incident heart failure: The atherosclerosis risk in communities (ARIC) study. Circulation Conference: American Heart Association's 2015;10.

18. Kurl S, Jae SY, Zaccardi F, Kauhanen J, Ronkainen K, Laukkanen JA. Exercise cardiac power and the risk of heart failure in men. European Heart Journal Conference: European Society of Cardiology, ESC Congress 2015;01.

19. Kupsky DF, Ahmed AM, Sakr S et al. Cardiorespiratory fitness and incident heart failure: The fit (henry ford exercise testing) project. Circulation Conference: American Heart Association's 2016;November.

20. LaMonte MJ, Larson JC, Manson JE et al. Physical activity and heart failure incidence among postmenopausal women in the women's health initiative study. Circulation Conference: American Heart Association's 2016;November.

21. Eaton CB, Larson JC, Chomistek AK et al. Physical activity and risk of heart failure with preserved or reduced ejection fraction in the women's health initiative study. Circulation Conference: American Heart Association's 2016;November.

22. Spahillari A, Talegawkar S, Correa A et al. Ideal cardiovascular health, cardiometabolic remodeling, and heart failure in African-Americans: The Jackson heart study. Circulation Conference: American Heart Association's 2016;November.

23. Florido R, Kwak L, Lazo M et al. Physical activity and incident heart failure in high-risk subgroups. Circulation Conference: American Heart Association's 2016;November.

24. Pandey A, LaMonte M, Klein L et al. Associations of physical activity levels, body mass index, and risks of heart failure with preserved ejection fraction vs. reduced ejection fraction: An individual-level pooled analysis. Circulation Conference: American Heart Association's 2016;November.

25. Lear S, Gasevic D, Hu W et al. The effect of overall and types of physical activity on mortality and cardiovascular events in 17 countries: Results from the prospective urban rural epidemiologic (Pure) study. Global Heart Conference: World Congress of Cardiology Scientific Sessions 2016;June.

26. Koo P, Gjelsvik A, Choudhary G et al. Prospective association of physical activity and heart failure hospitalizations among african-american adults with normal ejection fraction: The Jackson heart study. Circulation Conference: American Heart Association's Epidemiology and Prevention/Lifestyle and Cardiometabolic Health 2016;01.

27. Djousse L, Chae C, Buring JE, Lee I-M. Usual walking pace is more strongly associated with lower risk of heart failure than total physical activity in the women's health study. Circulation Conference: American Heart Association's Epidemiology and Prevention/Lifestyle and Cardiometabolic Health 2016;01.

28. Ogunmoroti O, Oni E, Allen NB et al. Life's simple 7 and incident heart failure: The multi-ethnic study of atherosclerosis. Circulation Conference: American Heart Association's Epidemiology and Prevention/Lifestyle and Cardiometabolic Health 2016;01.

29. Khan H, Kella D, Kunutsor SK et al. Long-term change in cardiorespiratory fitness in relation to risk of incident heart failure and atrial fibrillation. Circulation Conference: Resuscitation Science Symposium, ReSS 2017;November.

30. Khan H, Kurl S, Laukkanen JA. Exercise cardiac power and risk of incident heart failure. Journal of the American College of Cardiology Conference: 66th Annual Scientific Session of the American College of Cardiology and i2 Summit: Innovation in Intervention, ACC 17 United States 2017;69 (11 Supplement 1): 942.

31. Akintoye E, Mahmoud K, Weinberger J, Afonso L. Left ventricular mass index and other independent predictors of incident heart failure. Journal of the American College of Cardiology Conference: 66th Annual Scientific Session of the American College of Cardiology and i2 Summit: Innovation in Intervention, ACC 17 United States 2017;69 (11 Supplement 1): 737.

32. Simek S, Pandey A, Ayers C et al. Physical activity, chronic myocardial injury and risk of heart failure in african americans: The jackson heart study. Circulation Conference: 2018;November.

33. Kokkinos PF, Narayan P, Faselis C et al. Obesity, heart failure risk & cardiorespiratory fitness. Circulation Conference: American Heart Association's Epidemiology and Prevention/Lifestyle and Cardiometabolic Health 2018;March.

34. Khan SS, Paluch AE, Ning H et al. Association of physical inactivity and lifetime risk of heart failure among white and black US adults. Circulation Conference: American Heart Association's Epidemiology and Prevention/Lifestyle and Cardiometabolic Health 2019;March.

35. Al-Ramady O, Chen J, Gaziano JM, Djousse L. Walking pace is inversely associated with heart failure risk in the physicians' health study. Circulation Conference: American Heart Association's Epidemiology and Prevention/Lifestyle and Cardiometabolic Health 2019;March.

36. LaMonte MJ, Bellettiere J, Eaton CB, Stefanick ML, LaCroix AZ. Accelerometer-measured physical activity and heart failure incidence in women ages 63-99 years: The opach study. Circulation Conference: American Heart Association's Epidemiology and Prevention/Lifestyle and Cardiometabolic Health 2019;March.

37. Bundy JD, Ning H, Paluch AE et al. Ideal cardiovascular health and lifetime risk of cardiovascular disease: The cardiovascular disease lifetime risk pooling project. Circulation Conference: American Heart Association's Epidemiology and Prevention/Lifestyle and Cardiometabolic Health 2019;March.

38. Hamo CE, Kwak L, Florido R et al. Heart failure risk associated with optimal levels of modifiable HF risk factors: The atherosclerosis risk in communities study (ARIC). Circulation Conference: American Heart Association's Epidemiology and Prevention/Lifestyle and Cardiometabolic Health 2019;March.

39. Noel CA, LaMonte MJ, Pearlman D et al. Healthy lifestyle and risk of incident heart failure with preserved and reduced ejection fraction in postmenopausal women: The women's health initiative study. Circulation Conference: American Heart Association's Epidemiology and Prevention/Lifestyle and Cardiometabolic Health 2019;March.

40. Pittaras A, Faselis C, Doumas M et al. Cardiorespiratory fitness and heart failure incidence in hypertensive patients. Journal of Hypertension Conference: 29th European Meeting on Hypertension and Cardiovascular Protection, ESH 2019;July.

41. Kokkinos P, Narayan P, Mordini F, Myers J, Ahmed A, Faselis C. Cardiorespiratory fitness attenuates the risk of congestive heart failure in hypertensives. Cardiology (Switzerland) Conference: 22nd World Congress on Heart Disease International Academy of Cardiology Annual Scientific Sessions 2017;2017.

42. Kokkinos P, Faselis C, Myers J, Narayan P. Exercise capacity and primary prevention of major adverse cardiac events in veterans: The ethos study. Circulation Conference: American

Heart Association's Epidemiology and Prevention/Lifestyle and Cardiometabolic Health 2016;01.

43. Jakicic JM, Fulton JE, Lang W, Walkup MP. Association of the change in physical activity and cardiovascular disease outcomes in the look ahead trial. Circulation Conference: American Heart Association's Epidemiology and Prevention/Lifestyle and Cardiometabolic Health 2018;March.

44. Lagat DK, DeLong AK, Wellenius GA et al. Factors associated with isolated right heart failure in women: a pilot study from western Kenya. Glob Heart 2014;9:249-54.

45. Maessen MF, Verbeek AL, Bakker EA, Thompson PD, Hopman MT, Eijsvogels TM. Lifelong Exercise Patterns and Cardiovascular Health. Mayo Clin Proc 2016;91:745-54.

46. Caron A, Ayala A, Damian J et al. Physical activity, body functions and disability among middle-aged and older Spanish adults. BMC Geriatr 2017;17:150.

47. Vasankari V, Husu P, Vaha-Ypya H et al. Association of objectively measured sedentary behaviour and physical activity with cardiovascular disease risk. European Journal of Preventive Cardiology 2017;24:1311-8.

48. Henriksson H, Henriksson P, Tynelius P et al. Cardiorespiratory fitness, muscular strength, and obesity in adolescence and later chronic disability due to cardiovascular disease: a cohort study of 1 million men. Eur Heart J 2020;14(15):1503-1510.

49. Wang Y, Tuomilehto J, Jousilahti P et al. Lifestyle factors in relation to heart failure among Finnish men and women. Circ Heart Fail 2011;4:607-12.

50. deFilippi CR, de Lemos JA, Tkaczuk AT et al. Physical activity, change in biomarkers of myocardial stress and injury, and subsequent heart failure risk in older adults. J Am Coll Cardiol 2012;60:2539-47.

51. Farrell SW, Finley CE, Radford NB, Haskell WL. Cardiorespiratory fitness, body mass index, and heart failure mortality in men: Cooper Center Longitudinal Study. Circ Heart Fail 2013;6:898-905.

52. Khan H, Kunutsor S, Rauramaa R et al. Cardiorespiratory fitness and risk of heart failure: a population-based follow-up study. Eur J Heart Fail 2014;16:180-8.

53. Andersen K, Rasmussen F, Held C, Neovius M, Tynelius P, Sundstrom J. Exercise capacity and muscle strength and risk of vascular disease and arrhythmia in 1.1 million young Swedish men: cohort study. BMJ 2015;351:h4543.

54. Abdelmawgoud A, Brown CJ, Sui X et al. Relationship of Physical Activity and Healthy Eating with Mortality and Incident Heart Failure among Community-Dwelling Older Adults with Normal Body Mass Index. ESC Heart Fail 2015;2:20-4.

55. Larsson SC, Tektonidis TG, Gigante B, Akesson A, Wolk A. Healthy lifestyle and risk of heart failure. Circulation: Heart Failure 2016;9:e002855.

56. Pandey A, Cornwell WK, III, Willis B et al. Body Mass Index and Cardiorespiratory Fitness in Mid-Life and Risk of Heart Failure Hospitalization in Older Age: Findings From the Cooper Center Longitudinal Study. JACC Heart Fail 2017;5:367-74.

57. Spahillari A, Talegawkar S, Correa A et al. Ideal Cardiovascular Health, Cardiovascular Remodeling, and Heart Failure in Blacks: The Jackson Heart Study. Circ Heart Fail 2017;10.

58. Lindgren M, Aberg M, Schaufelberger M et al. Cardiorespiratory fitness and muscle strength in late adolescence and long-term risk of early heart failure in Swedish men. Eur J Prev Cardiol 2017;24:876-84.

59. Khan H, Jaffar N, Rauramaa R, Kurl S, Savonen K, Laukkanen JA. Cardiorespiratory fitness and nonfatalcardiovascular events: A population-based follow-up study. Am Heart J 2017;184:55-61.

60. Tikkanen E, Gustafsson S, Ingelsson E. Associations of Fitness, Physical Activity, Strength, and Genetic Risk With Cardiovascular Disease: Longitudinal Analyses in the UK Biobank Study. Circulation 2018;137:2583-91.

61. Sillars A, Celis-Morales CA, Ho FK et al. Association of Fitness and Grip Strength With Heart Failure: Findings From the UK Biobank Population-Based Study. Mayo Clin Proc 2019;94:2230-40.

62. Cuthbertson CC, Tan X, Heiss G et al. Associations of Leisure-Time Physical Activity and Television Viewing With Life Expectancy Free of Nonfatal Cardiovascular Disease: The ARIC Study. J Am Heart Assoc 2019;8:e012657.

63. Schmidt M. Interaction between body mass index and physical fitness in primary heart failure prevention. Heart 2017;103:1749.

64. Ahmad T, Testani JM. Physical Activity Prevents Obesity and Heart Failure: Now What Are We Going to Do About It? JACC: Heart Failure 2017;5:385-7.

65. Leggio M, Tiberti C, Armeni M, Limongelli G, Mazza A. The Importance of Baseline Physical Activity Level in Heart Failure With Preserved Ejection Fraction. JACC: Heart Failure 2019;7:534-5.

66. Pandey A, Garg S, Khunger M et al. Dose-Response Relationship Between Physical Activity and Risk of Heart Failure: A Meta-Analysis. Circulation 2015;132:1786-94.

67. Echouffo-Tcheugui JB, Butler J, Yancy CW, Fonarow GC. Association of Physical Activity or Fitness With Incident Heart Failure: A Systematic Review and Meta-Analysis. Circ Heart Fail 2015;8:853-61.

68. Wahid A, Manek N, Nichols M et al. Quantifying the Association Between Physical Activity and Cardiovascular Disease and Diabetes: A Systematic Review and Meta-Analysis. J Am Heart Assoc 2016;5.

69. Qiu S, Cai X, Liu J et al. Association between cardiorespiratory fitness and risk of heart failure: a meta-analysis. J Card Fail 2019;25(7):537-544.

70. Djousse L, Driver JA, Gaziano JM. Relation between modifiable lifestyle factors and lifetime risk of heart failure. JAMA 2009;302:394-400.

71. Kubota Y, Evenson KR, Maclehose RF, Roetker NS, Joshu CE, Folsom AR. Physical Activity and Lifetime Risk of Cardiovascular Disease and Cancer. Med Sci Sports Exerc 2017;49:1599-605.

72. Folsom AR, Shah AM, Lutsey PL et al. American Heart Association's Life's Simple 7: Avoiding Heart Failure and Preserving Cardiac Structure and Function. Am J Med 2015;128:970-6.

73. Rariden BS, Boltz AJ, Brawner CA et al. Sedentary Time and Cumulative Risk of Preserved and Reduced Ejection Fraction Heart Failure: From the Multi-Ethnic Study of Atherosclerosis. Journal of Cardiac Failure 2019;25:418-24.

74. Hu G, Tuomilehto J, Silventoinen K, Barengo N, Jousilahti P. Joint effects of physical activity, body mass index, waist circumference and waist-to-hip ratio with the risk of cardiovascular disease among middle-aged Finnish men and women. Eur Heart J 2004;25:2212-9.

75. Carlsson AC, Arnlov J, Sundstrom J, Michaelsson K, Byberg L, Lind L. Physical activity, obesity and risk of cardiovascular disease in middle-aged men during a median of 30 years of follow-up. Eur J Prev Cardiol 2016;23:359-65.

76. Polonsky TS, Ning H, Daviglus ML et al. Association of Cardiovascular Health With Subclinical Disease and Incident Events: The Multi-Ethnic Study of Atherosclerosis. J Am Heart Assoc 2017;6(3):e004894.

77. Kubota Y, Iso H, Yamagishi K, Sawada N, Tsugane S. Daily Total Physical Activity and Incident Cardiovascular Disease in Japanese Men and Women: Japan Public Health Center-Based Prospective Study. Circulation 2017;135:1471-3.

78. Williams PT. Walking and running produce similar reductions in cause-specific disease mortality in hypertensives. Hypertension 2013;62:485-91.

79. Pandey A, LaMonte M, Klein L et al. Relationship Between Physical Activity, Body Mass Index, and Risk of Heart Failure. J Am Coll Cardiol 2017;69:1129-42.

80. Cohn JN. Prevention of heart failure. Cardiology 1999;92 Suppl 1:22-5.

81. Kokkinos PF, Choucair W, Graves P, Papademetriou V, Ellahham S. Chronic heart failure and exercise. Am Heart J 2000;140:21-8.

82. Pina IL, Apstein CS, Balady GJ et al. Exercise and heart failure: A statement from the American Heart Association Committee on exercise, rehabilitation, and prevention. Circulation 2003;107:1210-25.

83. Katz DL. Lifestyle and dietary modification for prevention of heart failure. Medical Clinics of North America 2004;88:1295-320.

84. Mirat J. [Physical activity in the prevention and treatment of cardiovascular diseases]. Acta Med Croatica 2007;61 Suppl 1:63-7.

85. Podolec P, Rubis P. [Heart failure and physical activity]. Przegl Lek 2007;64:86-90.

86. Wang Y, Hu G. Individual and joint associations of obesity and physical activity on the risk of heart failure. Congest Heart Fail 2010;16:292-9.

87. Lollgen H, Lollgen D. Risk reduction in cardiovascular diseases by physical activity. [German]. Internist 2011;1-8.

88. Thibault R, Meyer P, Cano N. Physical activity, nutrition, and chronic heart failure. [French]. Nutrition Clinique et Metabolisme 2014;28:327-35.

89. Gonzales AI, Sties SW, Mara LS, de CT. Influence of dance on cardiovascular health and sexual function. [Portuguese]. Revista Brasileira de Medicina 2015;72:161-5.

90. Pandey A, Darden D, Berry JD. Low Fitness in Midlife: A Novel Therapeutic Target for Heart Failure with Preserved Ejection Fraction Prevention. Progress in Cardiovascular Diseases 2015;58:87-93.

91. Hussain SR, Macaluso A, Pearson SJ. High-Intensity Interval Training Versus Moderate-Intensity Continuous Training in the Prevention/Management of Cardiovascular Disease. Cardiol Rev 2016;24:273-81.

92. Kondamudi N, Haykowsky M, Forman DE, Berry JD, Pandey A. Exercise Training for Prevention and Treatment of Heart Failure. Progress in Cardiovascular Diseases 2017;60:115-20.

93. Gong TA, Hall SA. Targeting Other Modifiable Risk Factors for the Prevention of Heart Failure: Diabetes, Smoking, Obesity, and Inactivity. Current Cardiovascular Risk Reports 2018;12:https://doi.org/10.1007/s12170-018-0574-9.

94. Rantanen T. Midlife fitness predicts less burden of chronic disease in later life. Clin J Sport Med 2013;23:499-500.

95. Amadid H, Johansen NB, Bjerregaard AL et al. The role of physical activity in the development of first cardiovascular disease event: a tree-structured survival analysis of the Danish ADDITION-PRO cohort. Cardiovasc Diabetol 2018;17:126.

96. Jefferis BJ, Parsons TJ, Sartini C et al. Does total volume of physical activity matter more than pattern for onset of CVD? A prospective cohort study of older British men. Int J Cardiol 2019;278:267-72.

97. Yusuf S, Joseph P, Rangarajan S et al. Modifiable risk factors, cardiovascular disease, and mortality in 155 722 individuals from 21 high-income, middle-income, and low-income countries (PURE): a prospective cohort study. Lancet 2020;395(10226):795-808.

98. Porter AK, Schilsky S, Evenson KR et al. The Association of Sport and Exercise Activities With Cardiovascular Disease Risk: The Atherosclerosis Risk in Communities (ARIC) Study. J Phys Act Health 2019;16(9):698-705.

Supplementary Table 2: Prospective studies of physical activity and cardiorespiratory fitness and heart failure incidence

Author, publication year, country/ region	Study name or description	Follow-up period	Study size, gender, age, number of cases	Exposure and subgroup	Quantity	RR (95% CI)	Adjustment for confounders
He J et al, 2001, USA	National Health and Nutrition Examination Survey Epidemiologic Follow-up Study	1971-1975 – 1992, 19 years follow-up	13643 men and women, age 25-74 years: 1382 congestive HF cases	Low physical activity, all<br>Low physical activity, men<br>Low physical activity, women	No vs. yes<br>No vs. yes<br>No vs. yes	1.22 (1.04-1.42)<br>1.14 (0.94-1.38)<br>1.31 (1.11-1.54)	Age, race, time-dependent history of CHD, sex (total), smoking, alcohol, hypertension, diabetes mellitus, valvular heart disease, overweight
Kenchaiah S et al, 2009, USA	Physicians' Health Study	1982-2007, 20.5 years follow-up	21094 men, age 40-84 years: 1109 HF cases	Vigorous physical activity<br><br><br><br>Vigorous physical activity<br><br><br><br>Vigorous physical activity	Rarely, never<br>1-3 times/month<br>1-4 times/week<br>5-7 times/week<br>Rarely, never<br>1-3 times/month<br>1-4 times/week<br>5-7 times/week<br>Rarely, never<br>1-3 times/month<br>1-4 times/week<br>5-7 times/week	1.00<br>0.77 (0.62-0.96)<br>0.80 (0.68-0.94)<br>0.64 (0.52-0.79)<br>1.00<br>0.76 (0.62-0.94)<br>0.82 (0.70-0.96)<br>0.69 (0.56-0.85)<br>1.00<br>0.78 (0.63-0.97)<br>0.86 (0.73-1.01)<br>0.73 (0.59-0.90)	Age, smoking status, alcohol, FH - MI, random assignment to aspirin or beta-carotene<br><br>+BMI<br><br><br><br>+hypertension, diabetes mellitus, hypercholesterolemia
Hu G et al, 2010, Finland	Finnish MONICA study	1972, 1977, 1982, 1987, 1992, 1997, 2002 - 2006, 18.4 years follow-up	59178 men and women, age 25-74 years: 1921/1693 HF cases	Total physical activity, men<br><br><br>Total physical activity, women	Low<br>Moderate<br>High<br>Low<br>Moderate<br>High	1.00<br>0.79 (0.68-0.92)<br>0.69 (0.60-0.80)<br>1.00<br>0.86 (0.75-0.99)<br>0.68 (0.59-0.78)	Age, study year, education, smoking, alcohol, history of MI, valvular heart disease, diabetes mellitus, SBP, total cholesterol, BMI
Wang Y et al, 2010, Finland	Finnish MONICA study	1972, 1977, 1982, 1987, 1992, 1997, 2002 - 2006, 18.4 years follow-up	28334 men and 29874 women, age 25-74 years: 1868/1640 HF cases	Occupational physical activity, men<br><br>Occupational, physical activity, women<br><br>Occupational physical activity, all	Low<br>Moderate<br>High<br>Low<br>Moderate<br>High<br>Low<br>Moderate	1.00<br>0.90 (0.78-1.03)<br>0.83 (0.73-0.93)<br>1.00<br>0.80 (0.70-0.92)<br>0.92 (0.82-1.05)<br>1.00<br>0.85 (0.77-0.93)	Age, study year, education, smoking, alcohol, history of MI, history of valvular heart disease, diabetes mellitus, SBP, total cholesterol, antihypertensive drugs, lung disease, BMI, mutual adjustment between physical activity types

					High	0.87 (0.80-0.94)	
				Commuting physical activity (walking/cycling), men	0 min/day<br>1-29<br>≥30	1.00<br>1.01 (0.90-1.13)<br>0.99 (0.87-1.12)	
				Commuting physical activity (walking/cycling), women	0 min/day<br>1-29<br>≥30	1.00<br>0.87 (0.76-0.99)<br>0.94 (0.82-1.07)	
				Commuting physical activity (walking/cycling), all	0 min/day<br>1-29<br>≥30	1.00<br>0.93 (0.85-1.01)<br>0.93 (0.85-1.02)	
				Leisure-time physical activity, men	Low<br>Moderate<br>High	1.00<br>0.83 (0.76-0.92)<br>0.65 (0.54-0.77)	
				Leisure-time physical activity, women	Low<br>Moderate<br>High	1.00<br>0.84 (0.75-0.94)<br>0.75 (0.60-0.94)	
				Leisure-time physical activity, all	Low<br>Moderate<br>High	1.00<br>0.83 (0.77-0.89)<br>0.69 (0.60-0.79)	
Bell EJ et al, 2013, USA	Atherosclerosis Risk in Communities Study	1987-2008, 17 years follow-up	3707 African American and 10018 Caucasian men and women, age 45-64 years: 633/1115 HF cases	Leisure-time physical activity, African Americans	0 min/week<br>1-149 min/week moderate or 1-74 min/week vigorous or 1-149 min/week of moderate/vigorous<br>≥150 min/week moderate or ≥75 min/week vigorous or ≥150 min/week moderate/vigorous	1.00<br>0.62 (0.51-0.75)<br>0.59 (0.47-0.74)	Age, sex, smoking, alcohol, HRT (women), education, Western and Prudent dietary pattern scores
				Leisure-time physical activity, Caucasians	0 min/week<br>1-149 min/week moderate or 1-74 min/week vigorous or 1-149 min/week of moderate/vigorous<br>≥150 min/week moderate or ≥75 min/week vigorous or ≥150 min/week moderate/vigorous	1.00<br>0.76 (0.65-0.88)<br>0.64 (0.54-0.75)	

Patel K et al, 2013, USA	Cardiovascular Health Study	1989-1990/1992-1993 - NA, 13 years follow-up	5503 men and women, age ≥65 years: 1137 HF cases	Leisure-time physical activity	0 MET-min/week<br>1-499<br>500-999<br>≥1000	1.00<br>0.97 (0.79-1.20)<br>0.86 (0.69-1.08)<br>0.79 (0.64-0.97)	Age, sex, race, education, income, alcohol, smoking, BMI, coronary artery disease, acute myocardial infarction, hypertension, diabetes mellitus, stroke, atrial fibrillation, left ventricular hypertrophy, left ventricular systolic dysfunction, systolic blood pressure, diastolic blood pressure, depression score, Mini-mental state examination score, serum cholesterol, serum albumin, serum creatinine, serum C-reactive protein
Kraigher-Krainer E et al, 2013, USA	Framingham Heart Study	1986-1990 - NA, 11.5 years follow-up	1142 men and women, age 67-97 years: 250 HF cases<br>108 HFpEF cases<br>106 HFrEF cases	Total physical activity index, all<br><br><br>Total physical activity index, all<br><br><br>Total physical activity index, HFpEF<br><br><br>Total physical activity index, HFpEF<br><br><br>Total physical activity index, HFrEF<br><br><br>Total physical activity index, HFrEF	24.9-30.0/24.2-29.8<br>30.1-34.3/29.9-33.7<br>34.5-63.2/33.8-57.6<br>24.9-30.0/24.2-29.8<br>30.1-34.3/29.9-33.7<br>34.5-63.2/33.8-57.6<br>24.9-30.0/24.2-29.8<br>30.1-34.3/29.9-33.7<br>34.5-63.2/33.8-57.6<br>24.9-30.0/24.2-29.8<br>30.1-34.3/29.9-33.7<br>34.5-63.2/33.8-57.6<br>24.9-30.0/24.2-29.8<br>30.1-34.3/29.9-33.7<br>34.5-63.2/33.8-57.6<br>24.9-30.0/24.2-29.8<br>30.1-34.3/29.9-33.7<br>34.5-63.2/33.8-57.6	1.00<br>0.75 (0.53-1.02)<br>0.61 (0.44-0.85)<br>1.00<br>0.84 (0.60-1.17)<br>0.65 (0.46-0.91)<br>1.00<br>0.45 (0.27-0.76)<br>0.60 (0.38-0.97)<br>1.00<br>0.54 (0.31-0.92)<br>0.66 (0.41-1.07)<br>1.00<br>0.93 (0.57-1.51)<br>0.68 (0.41-1.13)<br>1.00<br>1.07 (0.64-1.79)<br>0.69 (0.41-1.19)	Age, sex, SBP, hypertension treatment, diabetes, valve disease, alcohol, left ventricular hypertrophy<br>+ BMI<br><br>Age, sex, SBP, hypertension treatment, diabetes, valve disease, alcohol, left ventricular hypertrophy<br>+ BMI<br><br>Age, sex, SBP, hypertension treatment, diabetes, valve disease, alcohol, left ventricular hypertrophy<br>+ BMI
Berry JD et al, 2013, USA	Cooper Center Longitudinal Study	1999-2009 - NA, 6.5 years follow-up	20642 men and women, mean age 49 years: 1051 HF hospitalizations	Physical fitness, men<br><br><br><br>Physical fitness, women	8.4 METs<br>10.3<br>13.0<br>Per MET<br>6.4 METs<br>7.9<br>10.0<br>Per MET	1.00<br>0.60 (0.49-0.75)<br>0.31 (0.24-0.41)<br>0.79 (0.75-0.83)<br>1.00<br>0.53 (0.31-0.93)<br>0.38 (0.20-0.71)<br>0.81 (0.68-0.96)	Age, SBP, diabetes mellitus, smoking, total cholesterol, BMI

Young DR et al, 2014, USA	California Men's Health Study	2002-2003 – 2012, 7.8 years follow-up	82695 men, age 45-69 years: 3473 HF cases	Leisure-time physical activity, all<br><br><br>Leisure-time physical activity, non-Hispanic white<br><br>Leisure-time physical activity, Hispanic<br><br>Leisure-time physical activity, Asian<br><br>Leisure-time physical activity, Black<br><br>Leisure-time physical activity, other	0-470 MET-min/week<br>471-1584<br>≥1585 MET-min/week<br>0-470 MET-min/week<br>471-1584<br>≥1585 MET-min/week<br>0-470 MET-min/week<br>471-1584<br>≥1585 MET-min/week<br>0-470 MET-min/week<br>471-1584<br>≥1585 MET-min/week<br>0-470 MET-min/week<br>471-1584<br>≥1585 MET-min/week<br>0-470 MET-min/week<br>471-1584<br>≥1585 MET-min/week	1.52 (1.38-1.67)<br>1.15 (1.04-1.26)<br>1.00<br>1.56 (1.39-1.76)<br>1.13 (1.00-1.27)<br>1.00<br>1.61 (1.23-2.13)<br>1.45 (1.09-1.93)<br>1.00<br>1.45 (1.06-1.98)<br>1.22 (0.89-1.67)<br>1.00<br>1.29 (0.97-1.72)<br>1.12 (0.82-1.53)<br>1.00<br>1.56 (1.06-2.30)<br>1.10 (0.73-1.66)<br>1.00	Age, race/ ethnicity, education, income, BMI, smoking status, hypertension, diabetes mellitus, antihypertensive medications, HDL-cholesterol, fasting glucose, triglycerides, calories from fat, fruits, vegetables, alcohol
Saevereid HA et al, 2014, Denmark	Copenhagen City Heart Study	1976-1978 - 2003 - 2011, 19.4 years follow-up	18209 men and women, age 20-80 years: 1580 HF cases<br>9937 men and women, age 20-80 years: 542 HF cases (walking)	Leisure-time physical activity, men<br><br><br>Duration of walking<br><br><br><br>Speed of walking<br><br><br>Leisure-time physical activity, women<br><br>Duration of walking<br><br><br><br>Speed of walking	Sedentary<br>Light<br>Moderate/high<br>Never-<0.5 hour/day<br>0.5-1 hour/day<br>1-2<br>>2<br>Low<br>Moderate<br>High<br>Sedentary<br>Light<br>Moderate/high<br>Never-<0.5 hour/day<br>0.5-1 hour/day<br>1-2<br>>2<br>Low<br>Moderate<br>High	1.00<br>0.76 (0.63-0.91)<br>0.87 (0.72-1.06)<br>1.00<br>0.90 (0.62-1.29)<br>0.76 (0.52-1.12)<br>1.10 (0.77-1.60)<br>1.00<br>0.49 (0.36-0.66)<br>0.28 (0.16-0.47)<br>1.00<br>0.77 (0.64-0.92)<br>0.72 (0.57-0.90)<br>1.00<br>0.68 (0.46-1.03)<br>0.76 (0.52-1.12)<br>0.78 (0.51-1.20)<br>1.00<br>0.59 (0.43-0.80)<br>0.34 (0.20-0.58)	Age, smoking status, cigarettes per day, alcohol, education, household income, FH - CVD, atrial fibrillation, impaired lung function, diabetes mellitus

				Leisure-time physical activity, men and women, excluding ACS diagnosed during follow-up<br>Duration of walking<br><br><br><br>Speed of walking	Sedentary<br>Light<br>Moderate/high<br>Never-<0.5 hour/day<br>0.5-1 hour/day<br>1-2<br>>2<br>Low<br>Moderate<br>High	1.00<br>0.68 (0.58-0.81)<br>0.76 (0.64-0.91)<br>1.00<br>0.72 (0.52-0.99)<br>0.79 (0.57-1.09)<br>0.97 (0.70-1.34)<br>1.00<br>0.53 (0.41-0.69)<br>0.27 (0.17-0.43)	
Andersen K et al, 2014, Sweden	Sweden National March Cohort	1997 - 2010, 13.3 years follow-up	39805 men and women, age ≥20 years: 1545 HF cases<br>1033 non-ischemic HF cases	Leisure-time physical activity, HF of any cause<br><br><br><br><br>Leisure-time physical activity, non-ischemic HF cases<br><br><br><br><br>Leisure-time physical activity, HF of any cause, women<br><br><br><br><br>Leisure-time physical activity, non-ischemic HF cases<br><br><br><br><br>Leisure-time physical activity, HF of any cause, men<br><br><br><br><br>Leisure-time physical activity, non-ischemic HF cases	<1.2 MET-hours/day<br>1.2-1.9<br>1.9-3.0<br>3.0-4.7<br>>4.7<br><1.2 MET-hours/day<br>1.2-1.9<br>1.9-3.0<br>3.0-4.7<br>>4.7<br><1.2 MET-hours/day<br>1.2-1.9<br>1.9-3.0<br>3.0-4.7<br>>4.7<br><1.2 MET-hours/day<br>1.2-1.9<br>1.9-3.0<br>3.0-4.7<br>>4.7<br><1.2 MET-hours/day<br>1.2-1.9<br>1.9-3.0<br>3.0-4.7<br>>4.7<br><1.2 MET-hours/day<br>1.2-1.9<br>1.9-3.0<br>3.0-4.7	1.00<br>0.93 (0.79-1.09)<br>0.79 (0.67-0.94)<br>0.73 (0.60-0.89)<br>0.65 (0.53-0.81)<br>1.00<br>0.83 (0.67-1.03)<br>0.80 (0.66-0.98)<br>0.66 (0.53-0.81)<br>0.61 (0.48-0.78)<br>1.00<br>0.81 (0.64-1.03)<br>0.70 (0.54-0.90)<br>0.70 (0.54-0.91)<br>0.61 (0.44-0.84)<br>1.00<br>0.77 (0.58-1.01)<br>0.75 (0.56-1.02)<br>0.64 (0.45-0.90)<br>0.59 (0.40-0.86)<br>1.00<br>1.07 (0.86-1.32)<br>0.90 (0.72-1.12)<br>0.77 (0.58-1.03)<br>0.71 (0.54-0.93)<br>1.00<br>0.91 (0.69-1.22)<br>0.86 (0.65-1.14)<br>0.68 (0.49-0.93)	Age, sex, alcohol, BMI, diabetes mellitus, hypertension, smoking, snuff use, WHR

					>4.7	0.65 (0.45-0.90)	
				Total physical activity, HF of any cause	<30.5 MET-hours/day	1.00	
					30.5-33.9	0.82 (0.68-1.00)	
					33.9-39.1	0.87 (0.72-1.04)	
					39.1-48.7	0.87 (0.73-1.05)	
					>48.7	0.90 (0.76-1.06)	
				Total physical activity, non-ischemic HF cases	<30.5 MET-hours/day	1.00	
					30.5-33.9	0.84 (0.66-1.07)	
					33.9-39.1	0.87 (0.70-1.09)	
					39.1-48.7	0.86 (0.69-1.06)	
					>48.7	0.98 (0.80-1.19)	
				Total physical activity, HF of any cause, women	<30.5 MET-hours/day	1.00	
					30.5-33.9	0.90 (0.68-1.19)	
					33.9-39.1	0.85 (0.64-1.13)	
					39.1-48.7	0.89 (0.65-1.21)	
					>48.7	1.13 (0.86-1.49)	
				Total physical activity, non-ischemic HF cases	<30.5 MET-hours/day	1.00	
					30.5-33.9	0.91 (0.67-1.24)	
					33.9-39.1	0.76 (0.55-1.05)	
					39.1-48.7	0.80 (0.58-1.11)	
					>48.7	1.09 (0.81-1.47)	
				Total physical activity, HF of any cause, men	<30.5 MET-hours/day	1.00	
					30.5-33.9	0.75 (0.58-0.97)	
					33.9-39.1	0.86 (0.67-1.09)	
					39.1-48.7	0.84 (0.67-1.05)	
					>48.7	0.80 (0.65-0.98)	
				Total physical activity, non-ischemic HF cases	<30.5 MET-hours/day	1.00	
					30.5-33.9	0.76 (0.52-1.09)	
					33.9-39.1	0.96 (0.71-1.31)	
					39.1-48.7	0.89 (0.65-1.20)	
					>48.7	0.93 (0.71-1.22)	
Rahman I et al, 2014, Sweden	Swedish Mammography Cohort	1997-2011, 13 years follow-up	27985 women, mean age 61.1 years: 2402 HF hospitalizations and deaths	Total physical activity	<39 MET-hours/d	1.00	Age, education, smoking status, cigarettes per day, alcohol, FH - MI, history of stroke, history of angina, hypertension, diabetes mellitus, BMI, waist circumference +additionally mutually adjusted for types of activity
					39-42	0.88 (0.79-0.98)	
					43-46	0.76 (0.68-0.85)	
					>47	0.73 (0.65-0.82)	
				Walking/bicycling	≥20 vs. <20 min/d	0.71 (0.64-0.80)	
				Exercise	≥1 vs. <1 hour/week	0.83 (0.75-0.92)	
				Work occupation	Active vs. mostly sitting	0.93 (0.83-1.03)	
				Home/household work	≥1 vs. <1 hour/week	0.82 (0.70-0.97)	

Agha G et al, 2014, USA	Women's Health Initiative	1993-1998 - NA, 11 years follow-up	84537 women, age 50-71 years: 1826 HF cases	Leisure-time physical activity	0 min/week<br>1-149 min/week moderate or 1-74 min/week vigorous or 1-149 min/week of moderate/vigorous<br>≥150 min/week moderate or ≥75 min/week vigorous or ≥150 min/week moderate/vigorous	1.00<br>0.77 (0.67-0.87)<br>0.69 (0.61-0.79)	Age, race/ethnicity, BMI, smoking status, Alternate Health Eating Index
Pandey A et al, 2015, USA	Cooper Center Longitudinal Study	1999-2009 (baseline) - 2009, 6.5 years follow-up	8683 participants, mean age 49 years: NA cases	Change in cardiorespiratory fitness (mean: 4.2 years after baseline)	Per 1 MET	0.83 (0.74-0.93)	Age, sex, BMI, cholesterol, baseline DM, smoking, SBP, interval DM, hypertension, AMI, COPD, CKD, obesity, obstructive sleep apnea
Chen Y et al, 2015, USA	Health and Retirement Study	2003 - 2004-2008, ~3.5 years of follow-up	1142 men and women, age ≥65 years: 255 HF cases	Leisure-time physical activity (moderate or vigorous)	<600 MET-min/week<br>≥600	1.00<br>0.78 (0.58-1.07)	Age, sex, race, marital status, years of schooling, household income, BMI, total illness burden index score, low cognition, insulin use, oral diabetes medications, screening adherence, medication adherence
Del Gobbo LC et al, 2015, USA	Cardiovascular Health Study	1989-1990/ 1992/1993 - NA, 21.5 years follow-up	4490 men and women, age ≥65 years: 1380 HF cases	Walking pace	<2 mph<br>≥2	1.00<br>0.80 (0.71-0.90)	Age, sex, race, enrolment site, education, annual income, healthy diet pattern, smoking status, alcohol, BMI,
Rahman I et al, 2015, Sweden	Cohort of Swedish Men	1998-2012, 13 years follow-up	33012 men, mean age 59.8 years: 3609 HF cases	Total physical activity<br><br><br>Walking/bicycling<br>Exercise<br>Work occupation<br>Home/household work<br>Walking/bicycling at 30 years and currently	30 MET-hours/d<br>41<br>47<br>≥20 vs. <20 min/d<br>≥1 vs. <1 hour/week<br>Active vs. mostly sitting<br>≥1 vs. <1 hour/week<br><20 min/d/<20 min/d<br>≥20 min/d/<20 min/d<br><20 min/d/≥20 min/d<br>≥20 min/d/≥20 min/d	1.44 (1.24-1.68)<br>1.00<br>1.25 (1.03-1.53)<br>0.79 (0.72-0.87)<br>0.86 (0.79-0.94)<br>0.93 (0.85-1.01)<br>0.95 (0.89-1.02)<br>1.00<br>1.01 (0.92-1.32)<br>0.77 (0.58-1.02)<br>0.84 (0.69-1.03)	Age, education, smoking status, cigarettes per day, alcohol, FH - MI, history of stroke, history of angina, hypertension, diabetes mellitus, BMI<br>+ mutual adjustment between types of activity

Eaton CB et al, 2016, USA	Women's Health Initiative	1993-1998 - 2015, 13.2 years follow-up	42170 postmenopausal women, age 50-79 years: 1952 HF cases<br>1419 HF cases with data on ejection fraction	Leisure-time physical activity, whites, HFpEF<br><br><br><br>Leisure-time physical activity, whites, HFrEF<br><br><br><br>Leisure-time physical activity, African Americans, HFpEF<br><br><br><br>Leisure-time physical activity, African Americans, HFrEF<br><br><br><br>Leisure-time physical activity, Hispanics, HFpEF<br><br><br><br>Leisure-time physical activity, Hispanics, HFrEF	≤1.25 MET-hours/week<br>1.25-<6.25<br>6.25-<15.3<br>≥15.3<br>≤1.25 MET-hours/week<br>1.25-<6.25<br>6.25-<15.3<br>≥15.3<br>≤1.25 MET-hours/week<br>1.25-<6.25<br>6.25-<15.3<br>≥15.3<br>≤1.25 MET-hours/week<br>1.25-<6.25<br>6.25-<15.3<br>≥15.3<br>≤1.25 MET-hours/week<br>1.25-<6.25<br>6.25-<15.3<br>≥15.3<br>≤1.25 MET-hours/week<br>1.25-<6.25<br>6.25-<15.3<br>≥15.3	1.00<br>0.94 (0.74-1.20)<br>0.83 (0.65-1.07)<br>0.75 (0.57-0.98)<br>1.00<br>0.95 (0.68-1.34)<br>0.72 (0.50-1.03)<br>0.77 (0.53-1.12)<br>1.00<br>0.77 (0.52-1.13)<br>0.73 (0.48-1.11)<br>0.65 (0.41-1.03)<br>1.00<br>0.81 (0.49-1.33)<br>0.75 (0.44-1.27)<br>0.59 (0.32-1.09)<br>1.00<br>1.62 (0.53-4.12)<br>0.87 (0.29-2.60)<br>1.32 (0.46-3.82)<br>1.00<br>1.08 (0.30-3.88)<br>0.45 (0.08-2.47)<br>1.34 (0.30-6.08)	Age, study component, race/ ethnicity, income, education, diabetes, heart rate, MI, CHD other than MI, stroke, smoking, dyslipidemia, oophorectomy, cancer, BMI, smoking, hypertension, chronic lung disease, anemia, atrial fibrillation, beta-blocker use, aspririn use, hormone therapy, alcohol, insurance, interim CHD - not MI, interim DM, interim cancer
Koo P et al, 2017, USA	The Jackson Heart Study	2000-2004 - 2012, 5.5 years follow-up	4066 black men and women, age 21-95 years: 168 HF cases	Leisure-time physical activity	0 min/week<br>1-149 min/week moderate or 1-74 min/week vigorous<br>≥150 min/week moderate or ≥75 min/week vigorous	1.00<br>0.74 (0.52-1.07)<br><br>0.41 (0.22-0.74)	Age, sex, BMI, smoking status, hypertension, chronic obstructive pulmonary disease, coronary heart disease, atrial fibrillation, diabetes mellitus, chronic kidney disease
Ogunmoroti O et al, 2017, USA	Multi-Ethnic Study of Atherosclerosis	2000-2002 - NA, 12.2 years follow-up	6506 men and women, age 45-84 years: 262 HF cases	Leisure-time physical activity, all<br><br><br><br><br>Leisure-time physical activity, whites	No exercise<br>1-149 min/week moderate or 1-74 min/week vigorous<br>≥150 min/week moderate or ≥75 min/week vigorous<br>No exercise<br>1-149 min/week moderate or 1-74 min/week vigorous	1.00<br>0.96 (0.66-1.39)<br><br>0.72 (0.54-0.96)<br><br>1.00<br>0.95 (0.51-1.76)	Age, sex, race/ethnicity, education, income, health insurance

					≥150 min/week moderate or ≥75 min/week vigorous	0.76 (0.46-1.24)	
				Leisure-time physical activity, Chinese Americans	No exercise<br>1-149 min/week moderate or 1-74 min/week vigorous<br>≥150 min/week moderate or ≥75 min/week vigorous	1.00<br>1.33 (0.29-6.04)<br>1.39 (0.38-5.11)	
				Leisure-time physical activity, African Americans	No exercise<br>1-149 min/week moderate or 1-74 min/week vigorous<br>≥150 min/week moderate or ≥75 min/week vigorous	1.00<br>1.09 (0.55-2.17)<br>0.85 (0.49-1.45)	
				Leisure-time physical activity, Hispanics	No exercise<br>1-149 min/week moderate or 1-74 min/week vigorous<br>≥150 min/week moderate or ≥75 min/week vigorous	1.00<br>0.79 (0.38-1.63)<br>0.41 (0.22-0.74)	
Lear SA et al, 2017, International	The PURE study	2003-2010 - NA, 6.9 years follow-up	130843 men and women, age 35-70 years: 386 HF cases	Total physical activity	<600 MET-min/week<br>600-3000<br>≥3000	1.00<br>0.83 (0.63-1.11)<br>0.76 (0.58-1.01)	Age, sex, education, country income level, urban/rural residency, FH - CVD, smoking status, household/community and country clustering
				Total physical activity, BMI adjusted	<600 MET-min/week<br>600-3000<br>≥3000	1.00<br>0.83 (0.62-1.12)<br>0.81 (0.61-1.08)	
				Total physical activity, excluding first two years of follow-up	<600 MET-min/week<br>600-3000<br>≥3000	1.00<br>0.77 (0.56-1.06)<br>0.78 (0.57-1.06)	
Myers J et al, 2017, USA	Veterans Exercise Testing Study	1984-2014, 12.3 years follow-up	21080 men and women, mean age 58.3 years: 1902 HF cases	Cardiorespiratory fitness	4.3 METs<br>6.0<br>7.3<br>8.7<br>11.6	1.00<br>0.64 (0.56-0.72)<br>0.59 (0.52-0.67)<br>0.36 (0.31-0.42)<br>0.25 (0.21-0.31)	Age, sex, BMI, ethnic origin, beta-blockers, calcium-channel blockers, angiotensin-converting enzymes, angiotensin receptor blockers, aspirin, diuretics, lipid-lowering agents, hypoglycemic agents, smoking, hypertension, diabetes mellitus, chronic kidney disease, HIV/AIDS

Crump C et al, 2017, Sweden	Swedish Military Conscripts	1969-1997 - 2012, 28.4 years follow-up	1330610 men, age 18 years: 11711 HF cases	Aerobic fitness<br><br><br>Aerobic fitness	<240 watts<br>240-288<br>≥289<br><240 watts<br>240-288<br>≥289	1.82 (1.69-1.96)<br>1.27 (1.19-1.37)<br>1.00<br>1.45 (1.34-1.56)<br>1.20 (1.11-1.29)<br>1.00	Age, year of military conscription examination, muscular strength, height, weight, education, neighbourhood SES, FH - HF<br>+ hypertension, ischemic heart disease, valvular heart disease, diabetes mellitus
Kupsky DF et al, 2017, USA	Henry Ford Exercise Testing (FIT) Project	1991-2009, 6.8 years follow-up	66329 men and women, mean age 55 years: 4652 HF cases	Cardiorespiratory fitness	<6 METs<br>6-9<br>10-11<br>≥12<br>Per 1 MET	1.00<br>0.56 (0.50-0.63)<br>0.34 (0.29-0.40)<br>0.16 (0.12-0.21)<br>0.83 (0.81-0.85)	Age, sex, race, known coronary artery disease, atrial fibrillation, diabetes, hypertension, hypertension medication use, ACE inhibitor use, antiotensin 2 receptor blocker use, beta-blocker use, diuretic use, hyperlipidemia, lipid-lowering medication use, obesity, sedentary lifestyle, pulmonary medication use, indication for stress testing, current smoking status
Khan H et al, 2017, Finland	Kuopio Ischemie Heart Disease Study	1984-1989 - 2011, 19.1 years follow-up	2089 men, age 42-61 years: 221 nonfatal HF cases	Cardiorespiratory fitness	1<br>2<br>3<br>4<br>Per 1 MET	1.00<br>0.87 (0.62-1.21)<br>0.58 (0.39-0.86)<br>0.49 (0.30-0.80)<br>0.84 (0.78-0.91)	Age, systolic blood pressure, BMI, CVD, diabetes, smoking, alcohol, serum creatinine, physical activity, SES
Georgiopoulou VV et al, 2017, USA	Health, Aging, and Body Composition Study	1997-1998 - 2007-2008, 10 years follow-up	2245 men and women, mean age 73.6 years: 253 HF cases	Walking speed over first 20 meter<br>Distance covered by 2 minutes<br>Speed to walk 400 meter	Per m/s<br>Per m<br>Per m/s	0.45 (0.18-1.09)<br>0.998 (0.99-1.005)<br>0.51 (0.20-1.31)	Age, sex, race, BMI, standing height, leg height, smoking, steps to complete first 20 m, self-reported physical activity, prevalent CVD, pulmonary disease, diabetes mellitus, hypertension, depression, SBP, heart rate, electrocardigraphic abnormalities, blood glucose, serum levels of albumin, creatinine, cholesterol
LaMonte MJ et al, 2018, USA	Women's Health Initiative	1993-1998 - 2015, 14 years	137303 women, age 50-79 years: 2523 HF cases	Leisure-time physical activity, all	0.0 MET-hours/week<br>3.8<br>11.4	1.00<br>0.89 (0.79-1.00)<br>0.74 (0.66-0.84)	Age, race/ethnicity, education, income, smoking status, alcohol use, HRT, hysterectomy

		follow-up			26.0	0.65 (0.57-0.74)	
			35272 women:	Leisure-time physical activity,	0.0 MET-hours/week	1.00	
			451 HFrEF cases	HFpEF	3.5	0.93 (0.76-1.14)	
			734 HFpEF cases		11.0	0.70 (0.56-0.88)	
					25.8	0.68 (0.54-0.86)	
				Leisure-time physical activity,	0.0 MET-hours/week	1.00	
				HFrEF	3.5	0.81 (0.63-1.04)	
					11.0	0.59 (0.45-0.79)	
					25.8	0.68 (0.41-0.91)	
				Leisure-time physical activity, all	0.0 MET-hours/week	1.00	+ treated diabetes, treated
					3.8	0.89 (0.80-1.00)	hypertension, SBP, DBP, atrial
					11.4	0.81 (0.71-0.91)	fibrillation
					26.0	0.75 (0.66-0.86)	
				Leisure-time physical activity,	0.0 MET-hours/week	1.00	
				HFpEF	3.5	0.95 (0.77-1.16)	
					11.0	0.78 (0.62-0.98)	
					25.8	0.82 (0.65-1.03)	
				Leisure-time physical activity,	0.0 MET-hours/week	1.00	
				HFrEF	3.5	0.79 (0.61-1.01)	
					11.0	0.60 (0.45-0.79)	
					25.8	0.71 (0.54-0.95)	
				Mild intensity physical activity,	0.0 MET-hours/week	1.00	
				all cases	1.5	1.01 (0.89-1.16)	
					3.5	0.88 (0.76-1.01)	
					7.5	0.80 (0.69-0.93)	
				Moderate intensity physical	0.0 MET-hours/week	1.00	
				activity	2.3	0.84 (0.74-0.95)	
					4.5	0.83 (0.74-0.94)	
					11.3	0.86 (0.77-0.97)	
				Strenous activity	0.0 MET-hours/week	1.00	
					3.5	0.86 (0.74-0.99)	
					11.7	0.73 (0.62-0.87)	
					24.5	0.64 (0.53-0.77)	
				Walking	0.0 MET-hours/week	1.00	
					1.5	0.88 (0.81-0.98)	
					5.0	0.69 (0.62-0.77)	
					12.5	0.58 (0.50-0.66)	
				Mild intensity physical activity,	0.0 MET-hours/week	1.00	
				HFpEF	1.5	1.02 (0.80-1.31)	

					3.5	0.97 (0.75-1.26)	
					7.5	0.81 (0.61-1.08)	
				Moderate intensity physical activity	0.0 MET-hours/week	1.00	
					2.3	0.82 (0.65-1.03)	
					4.5	0.84 (0.66-1.07)	
					11.3	0.97 (0.78-1.21)	
				Strenous activity	0.0 MET-hours/week	1.00	
					3.5	0.83 (0.63-1.08)	
					11.7	0.87 (0.65-1.18)	
					24.5	0.65 (0.46-0.92)	
				Walking	0.0 MET-hours/week	1.00	
					1.5	0.93 (0.78-1.11)	
					5.0	0.68 (0.55-0.83)	
					12.5	0.69 (0.53-0.89)	
				Mild intensity physical activity, HFrEF	0.0 MET-hours/week	1.00	
					1.5	0.88 (0.62-1.24)	
					3.5	0.95 (0.68-1.33)	
					7.5	1.10 (0.80-1.52)	
				Moderate intensity physical activity	0.0 MET-hours/week	1.00	
					2.3	0.65 (0.47-0.89)	
					4.5	0.83 (0.62-1.12)	
					11.3	0.78 (0.58-1.06)	
				Strenous activity	0.0 MET-hours/week	1.00	
					3.5	0.93 (0.67-1.28)	
					11.7	0.71 (0.47-1.09)	
					24.5	1.03 (0.71-1.49)	
				Walking	0.0 MET-hours/week	1.00	
					1.5	1.01 (0.81-1.27)	
					5.0	0.82 (0.64-1.05)	
					12.5	0.61 (0.43-0.86)	
Florido R et al, 2018, USA	Atherosclerosis Risk in Communities Study	1987-1989 - 1993-1995 - 2013, 19 years follow-up	11351 men and women, age 45-64 years: 1750 HF cases	Changes in physical activity from baseline to third follow-up	Poor/poor	1.00	Age, race, sex, smoking status, alcohol use
					Poor/intermediate	0.90 (0.74-1.08)	
					Poor/recommended	0.77 (0.63-0.93)	
					Intermediate/poor	0.93 (0.77-1.12)	
					Intermediate/intermediate	0.82 (0.67-1.00)	
					Intermediate/recommended	0.78 (0.65-0.95)	
					Recommended/poor	0.80 (0.65-0.98)	
					Recommended/intermediate	0.76 (0.62-0.94)	
					Recommended/recommended	0.69 (0.60-0.80)	

				Changes in physical activity from baseline to third follow-up<br><br><br><br><br><br><br><br><br>Changes in physical activity from baseline to third follow-up	Poor/poor<br>Poor/intermediate<br>Poor/recommended<br>Intermediate/poor<br>Intermediate/intermediate<br>Intermediate/recommended<br>Recommended/poor<br>Recommended/intermediate<br>Recommended/recommended<br>512.5 MET-min increase/week<br>750 MET-min increase/week<br>1000 MET-min increase/week	1.00<br>0.93 (0.77-1.12)<br>0.80 (0.66-0.98)<br>1.01 (0.83-1.21)<br>0.92 (0.75-1.14)<br>0.90 (0.74-1.09)<br>0.85 (0.69-1.05)<br>0.89 (0.72-1.10)<br>0.82 (0.71-0.96)<br>0.89 (0.82-0.96)<br><br>0.84 (0.75-0.95)<br>0.79 (0.68-0.93)	+ systolic blood pressure, antihypertensive medication use, diabetes mellitus, LDL cholesterol, HDL cholesterol, triglycerides, BMI measured at visit 1
Uijl A et al, 2019, Netherlands	European Prospective Investigation into Cancer and Nutrition - Netherlands cohort	1993-1997 - 2011, 15.2 years follow-up	37803 men and women, mean age 20-64 years: 690 HF cases	Total physical activity	Inadequate<br>Intermediate<br>Ideal	1.00<br>0.88 (0.69-1.11)<br>0.85 (0.66-1.09)	Age, sex, education, blood glucose, smoking status, BMI, blood pressure, diet (fruit and vegetables, whole grains, sodium, sugar-sweetened beverages), cholesterol
Ng R et al, 2019, Canada	Canadian Community Health Survey	2001-2010 - 2014, ~8.5 years follow-up	112870 men and women, age ≥20 years: NA	Leisure-time physical activity, women<br><br><br><br>Leisure-time physical activity, men	0 METs/day<br>0-1.5<br>1.5-3.0<br>≥3.0<br>0 METs/day<br>0-1.5<br>1.5-3.0<br>≥3.0	1.57 (1.12-2.19)<br>1.07 (0.79-1.44)<br>1.13 (0.82-1.55)<br>1.00<br>1.11 (0.79-1.58)<br>0.91 (0.71-1.16)<br>0.75 (0.57-0.99)<br>1.00	Age, ethnicity, immigrant status, rural residence, education, income, marital status, self-rated health, life stress, asthma, high blood pressure
Uijl A et al, 2019, United Kingdom	CALIBER	2000-2010 - NA, 5.8 years follow-up	871687 men and women, age ≥55 years: 47987 HF cases	Leisure-time physical activity, age 55-64 years, men<br>Leisure-time physical activity, age 65-74 years, men<br>Leisure-time physical activity, age ≥75 years, men<br>Leisure-time physical activity, age 55-64 years, women<br>Leisure-time physical activity, age 65-74 years, women	Sedentary vs. active<br><br>Sedentary vs. active<br><br>Sedentary vs. active<br><br>Sedentary vs. active<br><br>Sedentary vs. active	1.06 (0.99-1.13)<br><br>1.11 (1.04-1.17)<br><br>1.09 (1.02-1.16)<br><br>1.09 (1.00-1.19)<br><br><br>1.09 (1.01-1.17)	Age, sex, ethnicity, blood-pressure lowering medication, lipid-regulating drugs

				Leisure-time physical activity, age ≥75 years, women	Sedentary vs. active	1.08 (1.02-1.15)	
Kokkinos P et al, 2019, USA	Exercise Testing and Health Outcomes Study	1987-2017 - 2017, 13.4 years follow-up	20254 men, mean age 58 years: 2979 HF cases	Cardiorespiratory fitness	Per 1 MET	0.84 (0.83-0.86)	Age, BMI, ethnicity, beta-blockers, calcium channel blockers, angiotensin-converting enzyme inhibitors, angiotensin receptor blockers, diuretics, lipid-lowering agents, hypoglycaemic agents, smoking status, type 2 diabetes, dyslipidaemia, hypertension
Sillars A et al, 2020, United Kingdom	UK Biobank	2007-2010 - 2015, 9 years follow-up	468941 men and women, age 37-73 years: 1812 HF cases<br><br>Aerobic fitness: 64364 participants: 156 HF cases	Leisure-time physical activity, women<br><br><br><br>Leisure-time physical activity, men<br><br><br><br>Aerobic fitness, women<br><br><br><br>Aerobic fitness, men	<802 MET-min/week<br>802-1737<br>1738-3386<br>>3386<br><802 MET-min/week<br>802-1737<br>1738-3386<br>>3386<br>1<br>2<br>3<br>4<br>1<br>2<br>3<br>4	1.88 (1.42-2.49)<br>1.28 (0.94-1.74)<br>1.14 (0.84-1.55)<br>1.00<br>1.36 (1.13-1.64)<br>1.07 (0.88-1.31)<br>0.94 (0.77-1.15)<br>1.00<br>3.58 (1.27-10.09)<br>2.37 (0.83-6.76)<br>0.71 (0.19-2.67)<br>1.00<br>4.41 (2.07-9.40)<br>2.16 (0.97-4.83)<br>0.95 (0.36-2.48)<br>1.00	Age, ethnicity, deprivation index, month of assignment, height, medication for CVD, prevalent comorbidity score

BMI=Body Mass Index, CHD= coronary heart disease, DBP=diastolic blood pressure, HF=heart failure, HFpEF=heart failure with preserved ejection fraction, HFrEF=heart failure with reduced ejection fraction, DM2=type 2 diabetes mellitus, FFQ=food frequency questionnaire, FH=family history, GL=glycemic load, HDL=high density lipoprotein cholesterol, HRT=hormone replacement therapy, IFG=impaired fasting glucose, Mg=magnesium, min=minutes, OC=oral contraceptive, PUFA=polyunsaturated fatty acids, SBP=systolic blood pressure, SFA=saturated fatty acids, TAG=triacylglycerol, trans FA = trans fatty acids, WHR=waist-to-hip ratio

Supplementary Table 3: Prospective studies of physical activity and cardiorespiratory fitness and heart failure mortality

Williams PT et al, 2013, USA	National Walkers' Health Study	1998-2001 - 2008, 9.6 years follow-up	8436 men and 33586 women, mean age 52.68 years: 53 heart failure deaths	Total exercise energy expenditure<br><br><br><br>Total exercise energy expenditure<br><br><br><br>Walking energy expenditure	<1.07 MET-hours/day<br>1.07-1.80<br>1.80-3.60<br>≥3.60<br><1.07 MET-hours/day<br>1.07-1.80<br>1.80-3.60<br>≥3.60<br><1.07 MET-hours/day<br>1.07-1.80<br>1.80-3.60<br>≥3.60	1.00<br>0.60 (0.27-1.31)<br>0.54 (0.26-1.11)<br>0.58 (0.24-1.37)<br>1.00<br>0.80 (0.33-1.94)<br>0.64 (0.28-1.44)<br>0.68 (0.25-1.88)<br>1.00<br>0.85 (0.62-1.18)<br>0.55 (0.39-0.78)<br>0.59 (0.37-0.94)	Age, age squared, race, sex, education, prior heart attack, aspirin use, red meat, fruit, alcohol<br><br>+medication use, BMI at baseline
Williams PT et al, 2013, USA	National Walkers' Health Study	1998-2001 - 2008, 9.4 years follow-up	7374 men and 31607 women, mean age 50.6 years: 36 HF deaths	Walking pace (HF -underlying cause of death)<br>Walking pace (HF - all related causes of death)	Increase per minute/mile<br><br>Increase per minute/mile	1.065 (1.024-1.107)<br><br>1.038 (1.017-1.059)	Age, sex, years of education, smoking status, prior history of a heart attack, aspirin use, walking energy expenditure, BMI, meat, fruit, alcohol
Hamer M et al, 2019, United Kingdom	The Health Survey for England and Scottish Health Surveys	1994-2008 - 2009, 9.4 years follow-up	65093 men and women, age ≥40 years: 259 HF deaths<br><br>59122 men and women (excluding prevalent CVD at baseline), age ≥40 years: 183 HF deaths	Meeting physical activity guidelines<br><br><br><br>Total physical activity<br><br><br><br><br>Total physical activity, excluding prevalent CVD at baseline	Inactive<br>Insufficient<br>Sufficient<br>High<br><1.64 MET-hours/week<br>1.65-9.37<br>9.38-19.30<br>19.31-37.60<br>>37.6<br><1.64 MET-hours/week<br>1.65-9.37<br>9.38-19.30<br>19.31-37.60<br>>37.6	1.00<br>0.90 (0.61-1.34)<br>0.67 (0.31-1.43)<br>0.62 (0.25-1.52)<br>1.00<br>0.68 (0.50-0.93)<br>0.35 (0.22-0.96)?<br>0.51 (0.33-0.78)<br>0.35 (0.20-0.63)<br>1.00<br>0.64 (0.44-0.93)<br>0.37 (0.22-0.64)<br>0.52 (0.32-0.85)<br>0.39 (0.21-0.74)	Age, sex, smoking, social occupational group, chronic illnesses, psychological distress

BMI =body mass index, HF= heart failure

Supplementary Table 4. Assessment of cardiorespiratory fitness

Author, publication year, country	Study	Method of assessment	Details of the assessment
Berry JD et al, 2013, USA & Pandey A et al, 2015, USA	Cooper Center Longitudinal Study	Maximal treadmill exercise (Balke protocol)	Treadmill speed is set at 88 m/min initially. In the first minute grade is set at 0%, then 2%, and an increase of 1% for every following minute up to 25 minutes, at which point the grade is unchanged. Speed is increased 5.4 m/min for each additional minute until the test is finished. The test was terminated when the participants reported exhaustion or by the physician for medical reasons. The test time using the protocol showed high correlations with directly measured maximal oxygen uptake (r = 0.92).
Myers J et al, 2017, USA	Veterans Exercise Testing Study	Symptom-limited treadmill test (ramp protocol/Bruce protocol)	An individualized ramp treadmill protocol was used at Palo Alto and a Bruce protocol in Washington DC. The degree of effort was assessed using the Borg 6-20 perceived exertion scale. Exercise capacity was estimated from peak treadmill speed and grade using the ramp protocol and exercise time using the Bruce protocol (VO_2 max=14.76–(1.379×time)+(0.451×time^2)–(0.012×time^3). The participants were encouraged until volitional fatigue unless there were other symptoms or indications for stopping.
Crump C et al, 2017, Sweden	Swedish Military Conscripts	Electrically braked stationary bicycle ergometer test (validated)	Fitness was assessed as the maximal aerobic workload in watts. There is a high correlation between maximal aerobic workload and maximal oxygen uptake (correlation of ~0.9 with VO_2 max) and the measurement is also highly reproducible (test–retest correlation of 0.95).
Kupsky DF et al, 2017, USA	Henry Ford Exercise Testing (FIT) Project	Exercise treadmill testing	Cardiorespiratory fitness was estimated from the workload achieved during the test and was expressed as metabolic equivalents (METs).
Khan H et al, 2017, Finland	Kuopio Ischemie Heart Disease Study	Electrically braked cycle ergometer test	Fitness was assessed by measuring maximal oxygen uptake using a respiratory gas exchange analyzer during the cycle ergometer test. The testing protocol included a 3-minute warm-up at 50 W followed by an increase in the workload by 20 W/min with the direct analyses of respiratory gases. The VO_2 max was defined as the highest value for the plateau of oxygen uptake and was also expressed as METs of oxygen consumption (One MET = 3.5 mL/kg per minute). Maximal exercise workload (in watts) was defined as the highest workload achieved during the exercise test.
Kokkinos P et al, 2019, USA	Exercise Testing and Health Outcomes Study	Maximal exercise treadmill test	Participants were encouraged to exercise until volitional fatigue unless there were other symptoms or indications for stopping.

			Exercise capacity (peak METs) for each participant was calculated by standardized American College of Sports Medicine equations based on treadmill speed and grade.
Sillars A et al, 2020, United Kingdom	UK Biobank	Bicycle ergometer test	Fitness was measured as maximum workload attained during the fitness test. The test consisted of three phases, a pre-test phase with resting ECG, an activity phase, with increasing cycling workload, and a recovery phase, where the participant stopped cycling and ECG is recorded.

Supplementary Table 5: Definition of heart failure across studies

Author, publication year, country/ region	Study name	ICD codes	Outcome definition
He J et al, 2001, USA	National Health and Nutrition Examination Survey Epidemiologic Follow-up Study	ICD-9: 428.0-428.9	Incident cases of congestive heart failure were defined as participants with one or more hospital or nursing home stays where the participant had a discharge diagnosis with an ICD-9 code of 428.0-428.9 or a death certificate report with underlying cause of death of ICD-9 code of 428.0-428.9.
Kenchaiah S et al, 2009, USA	Physicians' Health Study	Not reported	Self-reported diagnosis by physician participants which were validated using mailed questionnaire on symptoms, signs, laboratory investigations, list of current medications: 89% of the validation study responders were on current treatment for heart failure and/or met the Framingham Heart Study criteria for heart failure
Hu G et al, 2010, Finland	Finnish MONICA study	ICD-8: 427.00, 427.10<br>ICD-9: 428, 4029B, 4148A-X<br>ICD-10: I50, I11.0, I13.0, I13.2	Linkage to the Finnish Hospital Discharge Register and the National Social Insurance Institution's Register on reimbursement for heart failure drugs for nonfatal outcomes and linkage to Finnish Death Register for fatal outcomes for ICD-codes ICD-8: 427.00 and 427.10, ICD-9: 428, 4029B, 4148A-X, ICD-10: I50, I11.0, I13.0, I13.2. Diagnosis was made by treating physician based on clinical assessment, x-ray examination, and echocardiography.
Wang Y et al, 2010, Finland	Finnish MONICA study	ICD-8: 427.00, 427.10<br>ICD-9: 428, 4029B, 4148A-X<br>ICD-10: I50, I11.0, I13.0, I13.2	Linkage to the Finnish Hospital Discharge Register and the National Social Insurance Institution's Register on reimbursement for heart failure drugs for nonfatal outcomes and linkage to Finnish Death Register for fatal outcomes for ICD-codes ICD-8: 427.00, 427.10, ICD-9: 428, 4029B, 4148A-X and<br>ICD-10: I50, I11.0, I13.0, I13.2. Diagnosis was made by a specialist based on the diagnosis, etiology, drug treatment and echocardiography.
Bell EJ et al, 2013, USA	Atherosclerosis Risk in Communities Study	ICD-9: 428.0-428.9<br>ICD-10: I50	First occurrence of either a hospitalization that included a primary or secondary diagnosis of ICD-9 code of 428.0-428.9, a death certificate with ICD-9 code of 428 or ICD-10 code of I50 among underlying causes of death. Validated using physician review of hospital records (predictive value of 93% for acute decompensated heart failure and 97% for chronic heart failure.
Patel K et al, 2013, USA	Cardiovascular Health Study	Not reported	Self-report of physician-diagnosed heart failure, which was confirmed by review of medical records for symptoms, signs, medications, and other evidence of heart failure. Heart failure was centrally adjudicated by the Cardiovascular Health Study Events Committee.
Kraigher-Krainer E et al, 2013, USA	Framingham Heart Study	Not reported	Heart failure was defined as meeting two major or one major plus two minor of the Framingham criteria. Heart failure cases were identified by review of hospitalization records, medical records from outpatient visits, and from data gathered at routine

			biennial follow-up visits or annual telephonic health history updates, which were reviewed and adjudicated by a physician panel.
Berry JD et al, 2013, USA	Cooper Center Longitudinal Study	ICD-9: 428, 402.01, 402.11, 402.91, 404.01, 404.03, 404.11, 404.13, 404.91, 404.93.	Heart failure cases were identified from hospitalization records/inpatient claims data from the Center for Medicare and Medicaid Services with primary diagnosis of heart failure by ICD-9 codes 428, 402.01, 402.11, 402.91, 404.01, 404.03, 404.11, 404.13, 404.91,and 404.93.
Young DR et al, 2014, USA	California Men's Health Study	ICD-9: 402.X1, 404.X1, 404.X3, 428.XX	Heart failure cases were identified from electronic records using ≥1 hospitalization with an ICD-9 diagnosis code of heart failure 402.X1, 404.X1, 404.X3, 428.XX or ≥2 outpatient diagnoses of heart failure. Diagnoses were validated using Framingham Criteria (positive predictive value was 97%).
Saevereid HA et al, 2014, Denmark	Copenhagen City Heart Study	ICD-8: 425.99, 427.09-427.11, 427.19, 428.99 ICD-10: I11.0, I25.5, I42.0, I42.6, I42.9, I50.0-I50.9	Heart failure cases were identified by linkages to the Danish National Patient Registry and the Danish Register of Causes of Death and were defined as first hospital admission with a heart failure diagnosis or death from heart failure using ICD-8 codes 425.99, 427.09-427.11, 427.19, and 428.99 and ICD-10 codes I11.0, I25.5, I42.0, I42.6, I42.9, I50.0-I50.9.
Andersen K et al, 2014, Sweden	Sweden National March Cohort	ICD-10: I50.0-9, ICD-7: 434.1, 434.2, ICD-8: 427.0, 427.1 ICD-9: 428.A, 428.B, 428.X	Heart failure cases were identified by record linkages to the Swedish National Patient Register and the Swedish Cause of Death Register. ICD-10 codes I50.0-9, ICD-7 codes 434.1, 434.2, ICD-8 codes 427.0, 427.1, or ICD-9 codes 428.A, 428.B, 428.X were used to identify heart failure cases.
Rahman I et al, 2014, Sweden	Swedish Mammography Cohort	ICD-10: I50, I11.0.	Heart failure was defined as first incident heart failure hospitalization or death identified through linkage to the Swedish National Patient Register and the Swedish Cause of Death Register using ICD-10 codes I50 and I11.0.
Agha G et al, 2014, USA	Women's Health Initiative	Not reported	Heart failure cases were identified by self-report of hospitalizations and were ascertained by medical record abstraction and classified by trained adjudicators using standardized methodology. Heart failure diagnosis required physician diagnosis of new-onset or worsened congestive heart failure on the reported hospital admission and ≥1 of 4 criteria:<br>HF diagnosed by a physician and receiving medical treatment for HF; symptoms plus documentation in the current medical record of a history of an imaging procedure that showed impaired left ventricular systolic or diastolic function, pulmonary edema/congestion on chest radiograph on the current admission, dilated ventricle(s) or “poor” left ventricular or right ventricular function by echocardiography, radionuclide ventriculography, or other contrast ventriculography; or evidence of left ventricular diastolic dysfunction.

Pandey A et al, 2015, USA	Cooper Center Longitudinal Study	ICD-9: 428, 402.01, 402.11, 402.91, 404.01, 404.03, 404.11, 404.13, 404.91, 404.93	Heart failure cases were identified by linkage to hospitalization records and was defined by ICD-9 codes 428, 402.01, 402.11, 402.91, 404.01, 404.03, 404.11, 404.13, 404.91, 404.93.
Chen Y et al, 2015, USA	Health and Retirement Study	ICD-9: 428.xx, 398.91, 402.01, 402.11, 402.91, 404.11, 404.91	Physician-diagnosis of congestive heart failure obtained by Medicare claims data ICD-9 codes 428.xx, 398.91, 402.01, 402.11, 402.91, 404.11, 404.91
Del Gobbo LC et al, 2015, USA	Cardiovascular Health Study	Not reported	Heart failure cases were identified during annual study clinic examinations and telephone contact and were adjudicated by a centralized committee using outpatient and inpatient medical records, diagnostic tests, clinical consultations, and interviews. Confirmation of HF required: 1) diagnosis by a treating physician; 2) HF symptoms (shortness of breath, fatigue, orthopnea, or paroxysmal nocturnal dyspnea) plus signs (edema, rales, tachycardia, gallop rhythm, or displaced apical impulse) or supportive findings on echocardiography, contrast ventriculography, or chest radiography; and 3) medical therapy for HF, defined as diuretics plus either digitalis or a vasodilator.
Rahman I et al, 2015, Sweden	Cohort of Swedish Men	ICD-10: I50, I11.0.	Heart failure cases were identified by linkage to the Swedish National Patient Register and the Swedish Cause of Death Register and was defined as first incident heart failure hospitalization or death using ICD-10 codes I50 and I11.0.
Eaton CB et al, 2016, USA	Women's Health Initiative	Not reported	Heart failure cases were identified by self-report of a hospitalization and were adjudicated by trained adjudicators.<br>Heart failure with reduced ejection fraction was defined as heart failure with ejection fraction <45%, and heart failure with ejection fraction ≥45% was considered heart failure with preserved ejection fraction.
Koo P et al, 2017, USA	The Jackson Heart Study	Not reported	Heart failure hospitalizations and deaths were identified by telephone interviews, annual follow-up data, and hospital discharge records. Heart failure was adjudicated by trained medical personnel based in ICD-9 codes.
Ogunmoroti O et al, 2017, USA	Multi-Ethnic Study of Atherosclerosis	Not reported	Heart failure cases were identified by self-report in telephone interviews which collected information on hospital admissions, cardiovascular outpatient diagnoses and deaths. Self-reported diagnoses were verified from death certificates, medical records for all hospitalizations, and outpatient diagnoses and adjudicated by two physicians, and if the adjudicators did not come to agreement a morbidity and mortality classification committee made the final decision. Heart failure was defined as a combination of probable and definite heart failure, with probable heart failure defined as a diagnosis of heart failure made by a physician and medical treatment for heart failure, and definite heart failure, ≥1 additional objective criteria were required: pulmonary edema/congestion by chest X-ray, dilated ventricle or poor left ventricular function by echocardiography or ventriculography, or evidence of left ventricular diastolic dysfunction.

Lear SA et al, 2017, International	The PURE study	Not reported	Death due to heart failure in absence of myocardial infarction or other causes was attributed to fatal heart failure. The diagnosis of congestive heart failure required 2 of the 3 following criteria: a) Signs (rales, increased jugular venous pressure or ankle edema) or symptoms (nocturnal paroxysmal dyspnea, dyspnea at rest of ankle edema) of congestive heart failure, b) Radiological signs of pulmonary congestion, c) Treatment of heart failure with diuretics.
Myers J et al, 2017, USA	Veterans Exercise Testing Study	ICD-9: 428.0-428.43.	Heart failure was determined through review of Computerized Patient Record System and defined as a primary diagnosis of HF using ICD-9 codes 428.0-428.43.
Crump C et al, 2017, Sweden	Swedish Military Conscripts	ICD-8: 427.0–427.1<br>ICD-9: 402.01, 402.11, 402.91, 428<br>ICD-10: I11.0, I13.0, I13.2, I50	Heart failure cases were identified by linkage to the Swedish Hospital and Outpatient Registries and a heart failure cases was defined as at least one inpatient diagnosis or at least two outpatient diagnoses using ICD-8: 427.0–427.1; ICD-9: 402.01, 402.11, 402.91, 428; ICD-10: I11.0, I13.0, I13.2, I50.
Kupsky DF et al, 2017, USA	Henry Ford Exercise Testing (FIT) Project	ICD-9: 428.X	Heart failure cases were determined via administrative claims files from services provided by the Henry Ford physician group practice or reimbursed by the patients' health care insurer and a heart failure diagnosis was made if ICD-9 code 428.X was documented in 3 separate encounters.
Khan H et al, 2017, Finland	Kuopio Ischemie Heart Disease Study	ICD-10: I50.0-I50.9, I11, I42.0-I42.9	Heart failure cases were identified from hospital records and mediocolegal reports and included ICD-10 codes I50.0-I50.9, I11, I42.0-I42.9). A heart failure diagnosis was based on diagnostic guidelines of the European Society of Cardiology including symptoms, signs, laboratory investigations including N-terminal pro–b-type natriuretic peptide, and findings from chest radiography, ECG findings and echocardiography.
Georgiopoulou VV et al, 2017, USA	Health, Aging, and Body Composition Study	Not reported	Heart failure cases were derived from hospitalizations with all first admissions with an overnight stay confirmed as related to heart failure, using the criteria of the Cardiovascular Health Study, were considered as incident HF events. The criteria required heart failure diagnosis by a physician and treatment for heart failure. Briefly, heart failure was confirmed if there was documentation of symptoms and signs, supporting imaging findings, or medical therapy for heart failure, including at least a diuretic and a vasodilator or digitalis.
LaMonte MJ et al, 2018, USA	Women's Health Initiative	Not reported	Heart failure cases were identified by self-report and the diagnoses were adjudicated by trained physicians. For overall heart failure, a diagnosis of new-onset congestive heart failure at hospital admission and at least 1 of 4 clinical observations, were required. This method had strong agreement (kappa 0.79) comparing central and local adjudicated heart failure. Acute heart failure with an ejection fraction <45% or ≥45% was classified as heart failure with reduced ejection fraction and heart failure with preserved ejection fraction, respectively.

Florido R et al, 2018, USA	Atherosclerosis Risk in Communities Study	ICD-9: 428<br>ICD-10: I50	Heart failure cases were identified from linkages to hospitalization and death records with heart failure as underlying cause of death and was determined by ICD-9 code 428 in the early follow-up and ICD-10 code I50 for the later follow-up.
Uijl A et al, 2019, Netherlands	European Prospective Investigation into Cancer and Nutrition - Netherlands cohort	428, 402.01, 402.11, 402.91, 404.01, 404.03, 404.11, 404.13, 404.91, 404.93	Heart failure cases were identified by linkages to the Hospital Discharge Register and Cause of Death Register at Statistics Netherlands and included cases with underlying disease for hospitalization or underlying cause of death of heart failure with ICD-9 codes 428, 402.01-402.91, 404.01-404.91, 404.13, 404.93
Ng R et al, 2019, Canada	Canadian Community Health Survey	Not reported	Heart failure cases were identified using validated algorithms from hospital and physician data and cause of death data from vital statistics.
Uijl A et al, 2019, United Kingdom	CALIBER	ICD-10: I110, I130, I132, I260, I50<br><br>READ: G580400, G210.00, G210000, G210100, G211100, G21z100, G230.00, G232.00, G234.00, G1yz100, 1O1..00, 662W.00, 662p.00, 8B29.00, 8H2S.00, 9Or0.00, G400.00, G41z.11, G554000, G554011, G58..00, G58..11, G580.00, G580.11, G580.12, G580.13, G580.14, G580000, G580100, G580200, G580300, G581.00, G581.11, G581.13, G581000, G582.00, G58z.00, G58z.12, G5yy900, G5yyA00, R2y1000	Heart failure cases were identified by record linkage to the Clinical Practice Research Database and Hospital Episode Statistics and was defined in CPRD as a diagnosis of heart failure or chronic left ventricular dysfunction on echocardiagram with READ codes, and in HES by a record of heart failure with ICD-10 codes
Kokkinos P et al, 2019, USA	Exercise Testing and Health Outcomes Study	Not reported	Heart failure diagnoses were determined through review of the Veterans Affairs Computerized Patient Record System and defined as a primary diagnosis of HF using ICD coding.

Sillars A et al, 2020, United Kingdom	UK Biobank	ICD-10: I50.0, I50.2, I50.9	Heart failure cases were identified by linkages to hospital and death records with ICD-10 codes I50.0, I50.2, I50.9

Supplementary Table 6. Relative risks and 95% confidence intervals from nonlinear dose-response analysis

Total physical activity			Leisure-time physical activity			Vigorous physical activity		Cardiorespiratory fitness	
MET-hours/day	RR (95% CI) HF incidence	RR (95% CI) HF incidence and mortality	MET-hours/week	RR (95% CI) HF incidence	RR (95% CI) HF incidence and mortality	MET-hours/week	RR (95% CI) HF incidence	METs	RR (95% CI) HF incidence
0.7	1.00	1.00	0	1.00	1.00	0	1.00	3.5	1.00
5	0.88 (0.81-0.96)	0.88 (0.84-0.94)	5	0.84 (0.80-0.89)	0.87 (0.84-0.89)	5	0.83 (0.80-0.87)	4	1.00 (0.96-1.03)
10	0.76 (0.64-0.91)	0.77 (0.68-0.87)	10	0.75 (0.68-0.82)	0.77 (0.74-0.81)	10	0.74 (0.70-0.78)	6	0.98 (0.82-1.18)
15	0.67 (0.51-0.86)	0.67 (0.57-0.81)	15	0.70 (0.64-0.77)	0.72 (0.69-0.76)	15	0.69 (0.65-0.73)	8	0.95 (0.70-1.29)
20	0.59 (0.42-0.82)	0.60 (0.48-0.75)	20	0.69 (0.64-0.75)	0.70 (0.68-0.73)	20	0.66 (0.62-0.71)	10	0.82 (0.57-1.19)
25	0.53 (0.35-0.78)	0.54 (0.42-0.71)	25	0.70 (0.64-0.77)	0.70 (0.67-0.73)	25	0.64 (0.59-0.71)	12	0.63 (0.44-0.91)
30	0.48 (0.30-0.75)	0.50 (0.37-0.67)	30	0.71 (0.62-0.81)	0.70 (0.66-0.75)			13	0.55 (0.39-0.79)
35	0.44 (0.27-0.72)	0.46 (0.34-0.64)	35	0.72 (0.60-0.87)	0.71 (0.64-0.77)				
40	0.41 (0.25-0.69)	0.44 (0.31-0.61)							
45	0.39 (0.23-0.66)	0.41 (0.29-0.58)							
50	0.37 (0.22-0.63)	0.39 (0.27-0.56)							
$p_{nonlinearity}$	0.03	0.001	$p_{nonlinearity}$	0.001	<0.0001	$p_{nonlinearity}$	<0.0001	$p_{nonlinearity}$	0.003

Supplementary Table 7. Study quality scores for studies on total physical activity and heart failure

Author, publication year	Representative-ness	Selection of non-exposed cohort	Exposure ascertainment	Demonstration of outcome not present at start	Adjustment for one confounder	Adjustment for a second confounder	Assessment of outcome	Long enough follow-up	Loss to follow-up	Total
Hu, 2010	1	1	0	1	1	1	1	1	0	7
Kraigher-Krainer, 2013	1	1	1	1	1	1	1	1	0	8
Andersen, 2014	1	1	1	1	1	1	1	1	0	8
Rahman, 2014	1	1	1	1	1	1	1	1	0	8
Rahman, 2015	1	1	1	1	1	1	1	1	0	8
Lear, 2017	1	1	0	0	1	1	1	1	0	6
Uijl, 2019	1	1	0	1	1	1	1	1	0	7

Supplementary Table 8. Study quality scores for studies on leisure-time physical activity and heart failure

Author, publication year	Representative-ness	Selection of non-exposed cohort	Exposure ascertainment	Demonstration of outcome not present at start	Adjustment for one confounder	Adjustment for a second confounder	Assessment of outcome	Long enough follow-up	Loss to follow-up	Total
He, 2001	1	1	0	1	1	1	1	1	0	7
Wang, 2010	1	1	1	1	1	1	1	1	0	8
Bell, 2013	1	1	1	1	1	1	1	1	0	8
Patel, 2013	1	1	0	1	1	1	1	1	0	7
Saevereid, 2014	1	1	0	1	1	1	1	1	1	8
Young, 2014	1	1	0	1	1	1	1	1	0	7
Andersen, 2014	1	1	1	1	1	1	1	1	0	8
Rahman, 2014	1	1	1	1	1	1	1	1	0	8
Rahman, 2015	1	1	1	1	1	1	1	1	0	8
Chen, 2015	1	1	0	0	1	1	1	1	0	6
Koo, 2017	1	1	0	1	1	1	1	1	0	7
Ogunmoroti, 2017	1	1	0	1	1	1	1	1	0	7
LaMonte, 2018	1	1	1	1	1	1	1	1	0	8

Ng, 2019	1	1	0	1	1	1	1	1	0	7
Uijl, 2019	1	1	0	1	1	1	1	1	0	7
Sillars, 2020	1	1	0	1	1	1	1	1	0	7

Supplementary Table 9. Study quality scores for studies on cardiorespiratory fitness and heart failure

Author, publication year	Representative-ness	Selection of non-exposed cohort	Exposure ascertainment	Demonstration of outcome not present at start	Adjustment for one confounder	Adjustment for a second confounder	Assessment of outcome	Long enough follow-up	Loss to follow-up	Total
Berry, 2013	1	1	1	0	1	1	1	1	0	7
Myers, 2017	0	1	1	1	1	1	1	1	0	7
Crump, 2017	1	1	1	0	1	1	1	1	0	7
Kupsky, 2017	1	1	1	1	1	1	1	1	0	8
Khan, 2017	1	1	1	1	1	1	1	1	1	9
Sillars, 2020	1	1	1	1	1	1	1	1	0	8

Supplementary Figure 1. Funnel plot of total physical activity and heart failure

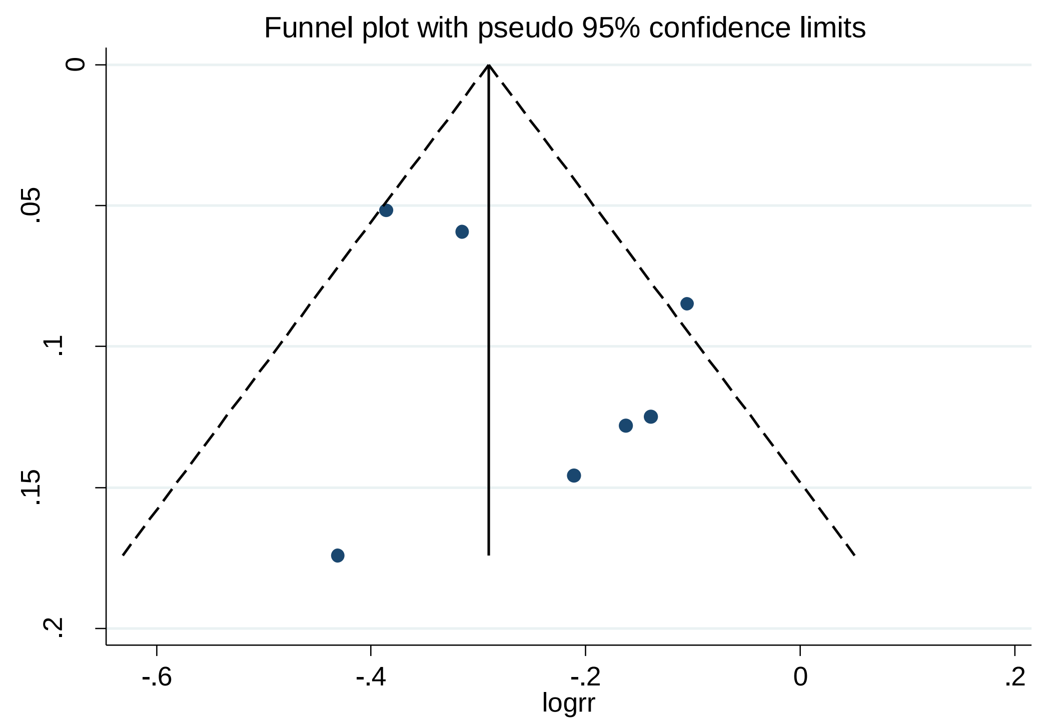

Supplementary Figure 2. Influence analysis of total physical activity and heart failure

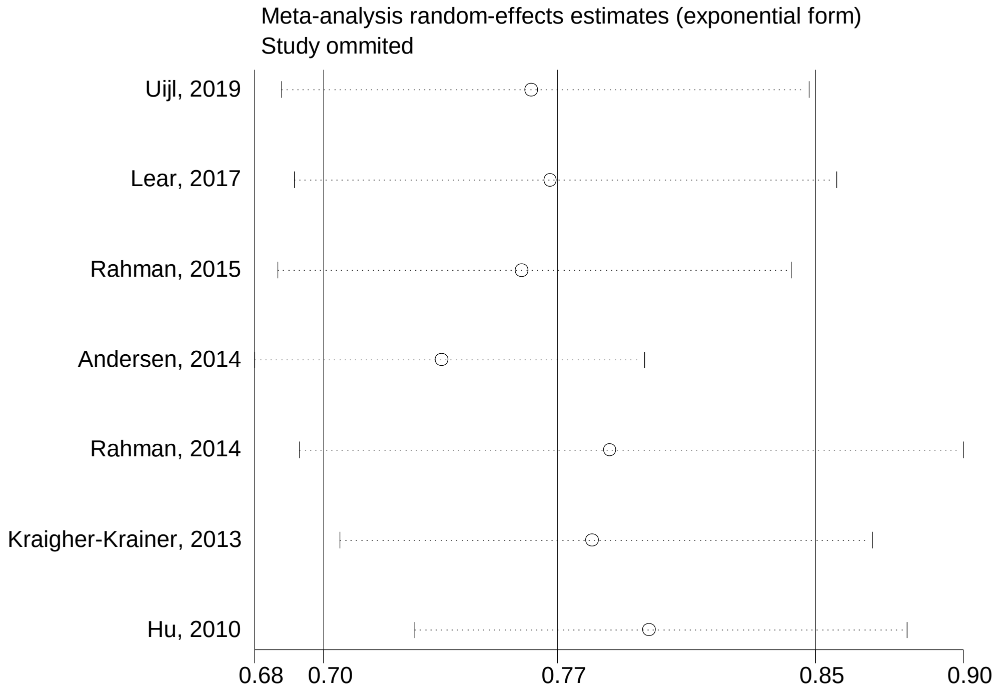

Study omitted	e^coef.	[95% Conf.	Interval]
Uijl, 2019	0.76255119	0.68578374	0.84791207
Lear, 2017	0.76850253	0.68970531	0.8563022
Rahman, 2015	0.75947964	0.68463224	0.84250975
Andersen, 2014	0.73501581	0.6776045	0.7972914
Rahman, 2014	0.78677696	0.69136411	0.89535737
Kraigher-Krainer, 2013	0.78129452	0.70378488	0.86734051
Hu, 2010	0.79882365	0.72671974	0.87808162
Combined	0.77062569	0.69875156	0.84989286

Supplementary Figure 3. Forest plot including one additional study on total physical activity and heart failure mortality (Hamer, 2019), high vs. low analysis

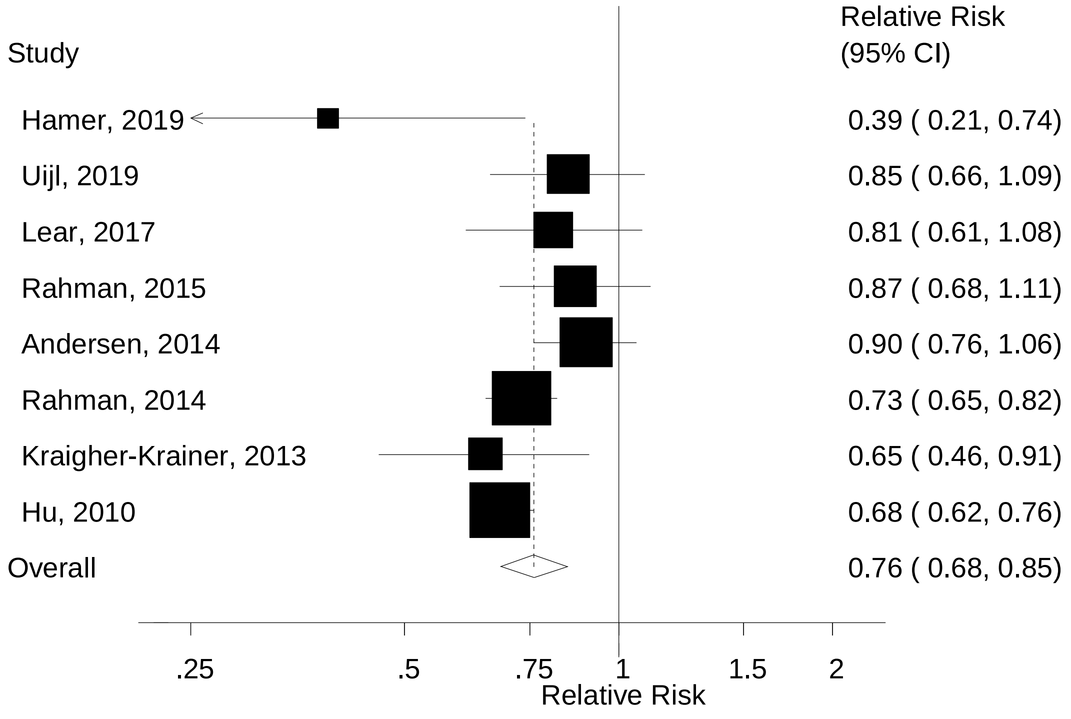

Supplementary Figure 4. Forest plot including one additional study on total physical activity and heart failure mortality (Hamer, 2019), dose-response analysis

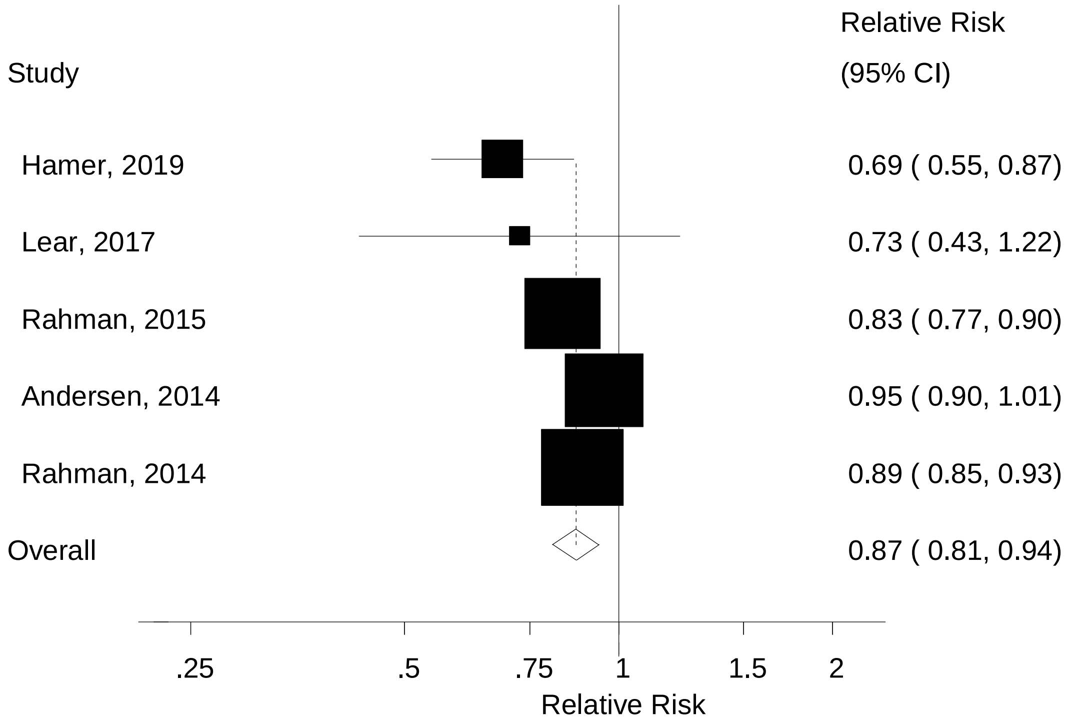

Supplementary Figure 5. Funnel plot of leisure-time physical activity and heart failure

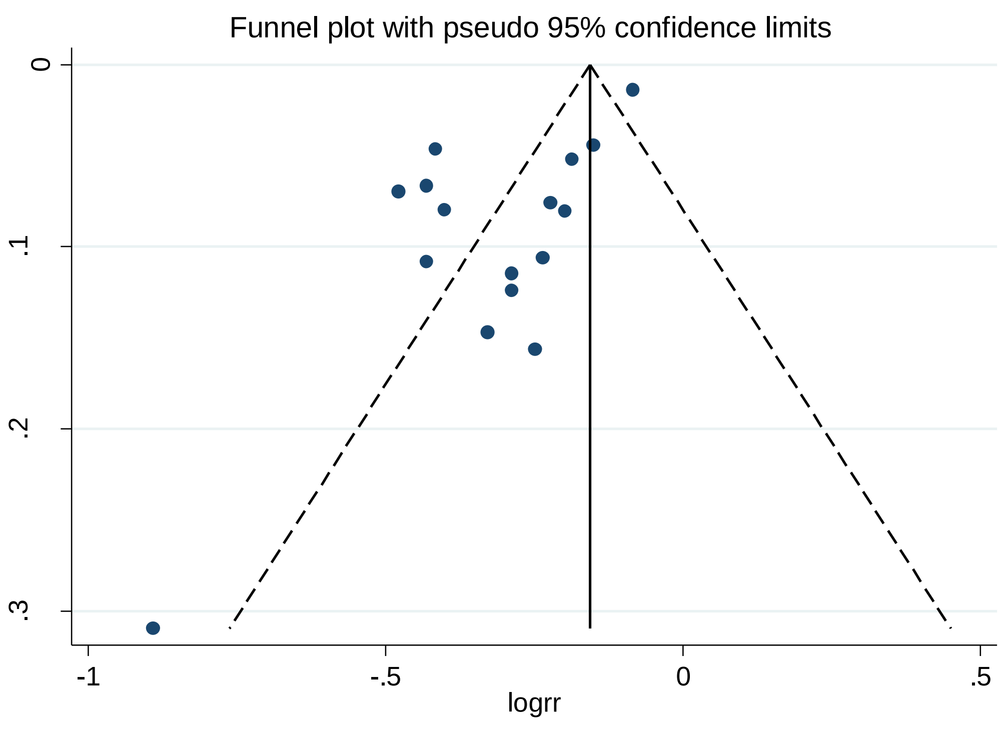

Supplementary Figure 6. Funnel plot of leisure-time physical activity and heart failure excluding the study by Uijl et al (2019).

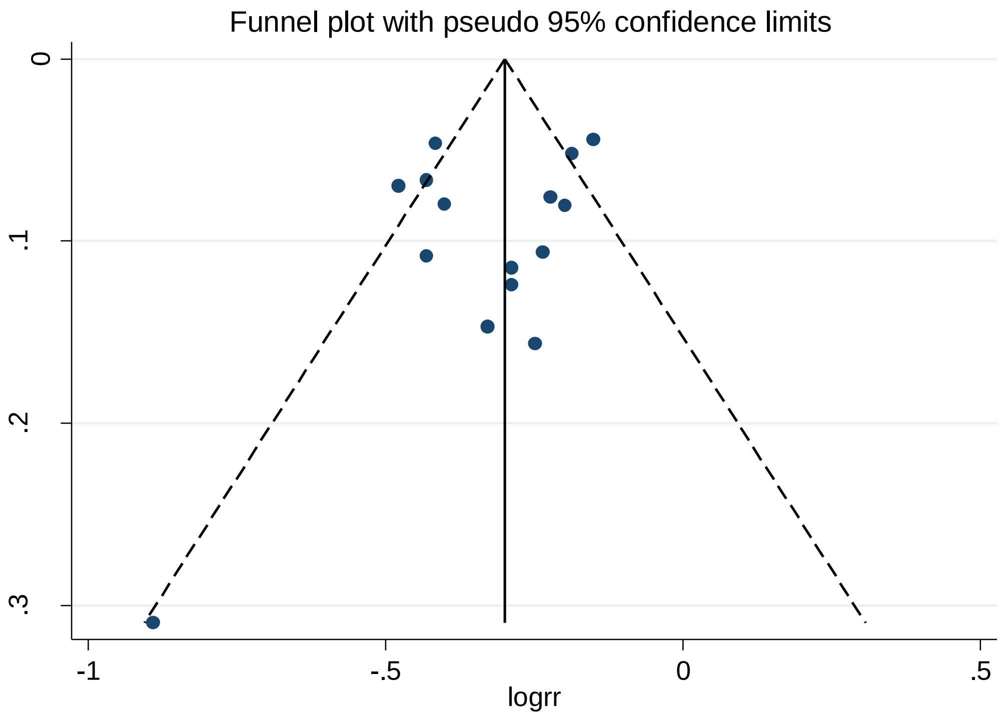

Supplementary Figure 7. Influence analysis of leisure-time physical activity and heart failure

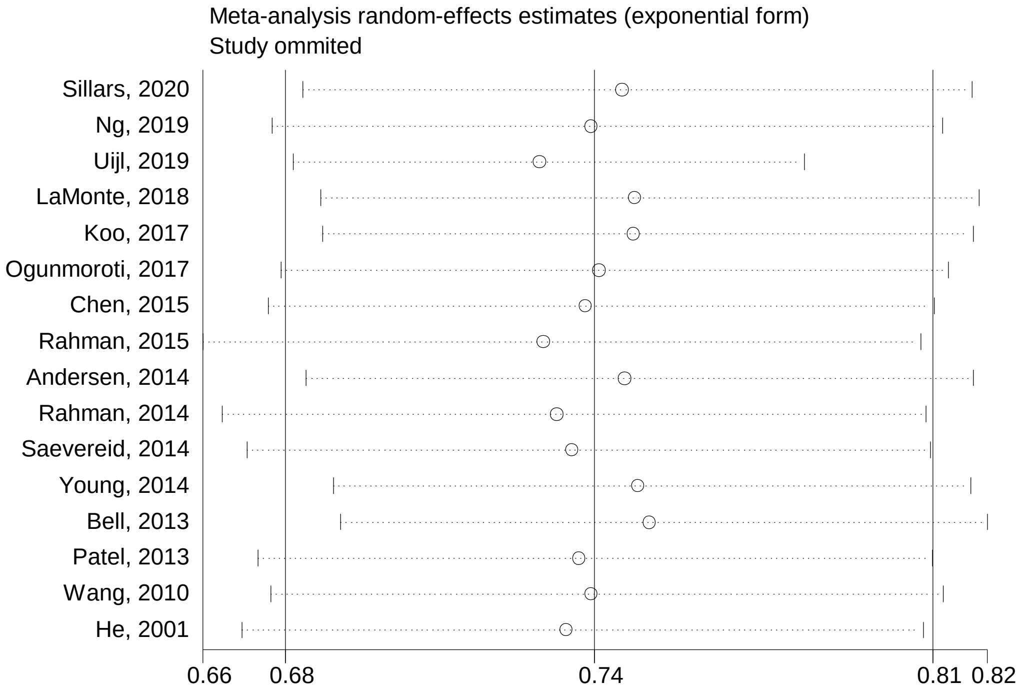

Study omitted	e^coef.	[95% Conf.	Interval]
Sillars, 2020	0.7471754	0.68067151	0.82017702
Ng, 2019	0.74081892	0.67418575	0.8140378
Uijl, 2019	0.72997653	0.67860889	0.78523254
LaMonte, 2018	0.74986029	0.68437767	0.82160836
Koo, 2017	0.74957937	0.6848219	0.82046038
Ogunmoroti, 2017	0.74241585	0.67608583	0.81525338
Chen, 2015	0.73959529	0.67341268	0.81228238
Rahman, 2015	0.73083776	0.65986526	0.80944389
Andersen, 2014	0.74767733	0.68134385	0.82046884
Rahman, 2014	0.73356766	0.66387224	0.81058002
Saevereid, 2014	0.73680204	0.66896826	0.81151426
Young, 2014	0.75054359	0.68702132	0.81993914
Bell, 2013	0.75295013	0.6885379	0.82338804
Patel, 2013	0.73824263	0.67130405	0.81185591
Wang, 2010	0.74077225	0.67398584	0.81417662
He, 2001	0.7355904	0.66796339	0.8100642
Combined	0.74145793	0.67698223	0.81207428

Supplementary Figure 8. Funnel plot of leisure-time physical activity and heart failure (dose-response analysis)

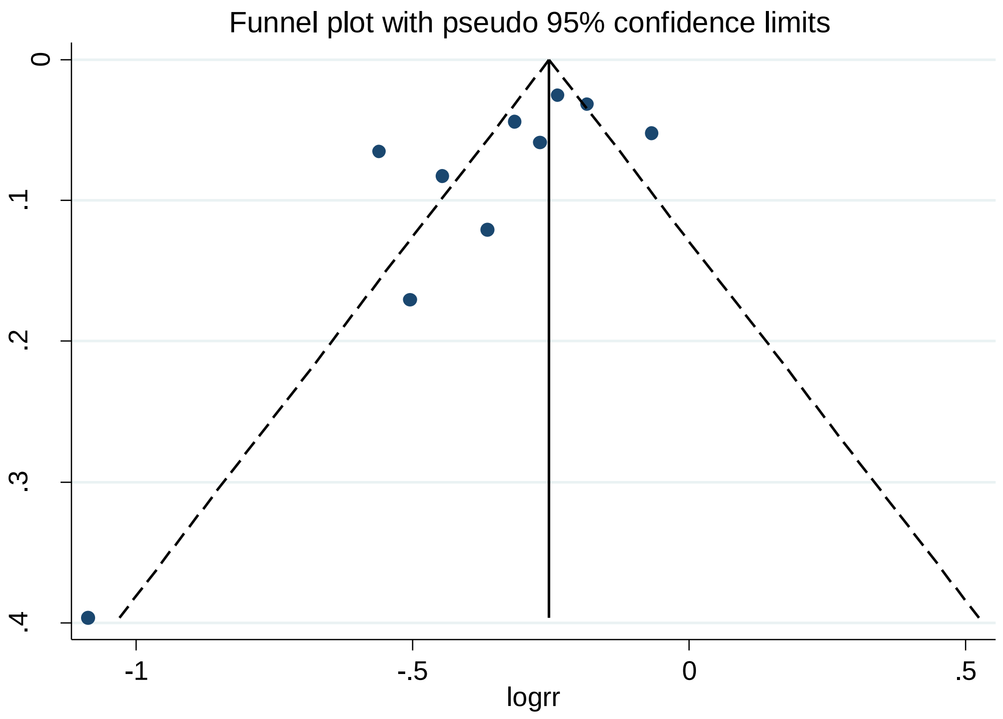

Supplementary Figure 9. Forest plot including one additional study on leisure-time physical activity and heart failure mortality (Williams, 2013), high vs. low analysis

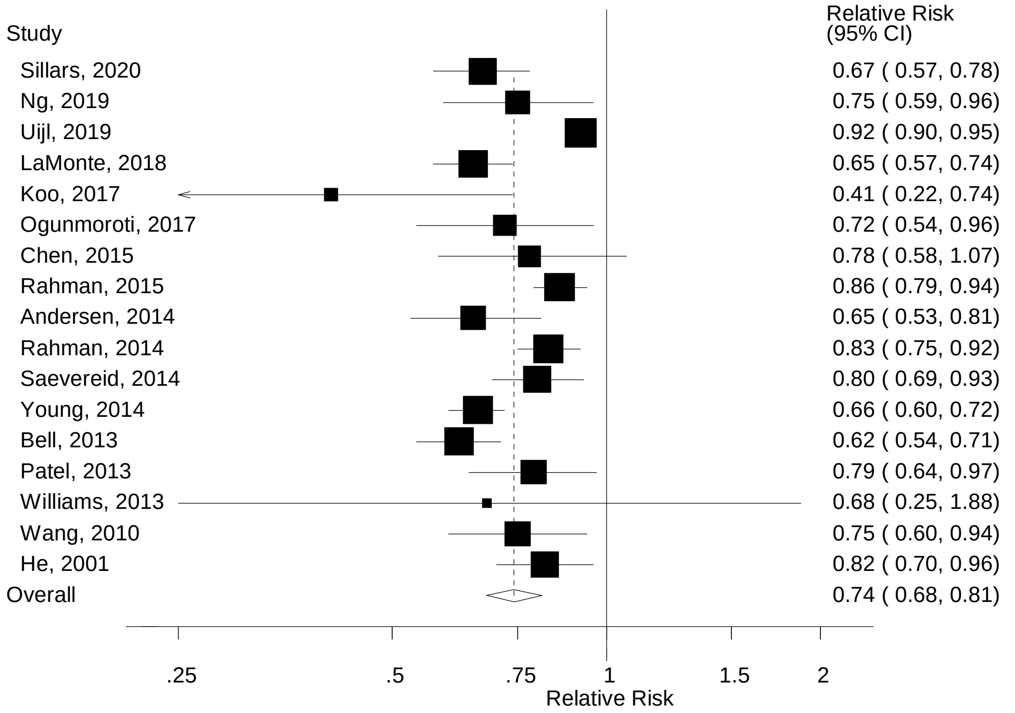

Supplementary Figure 10. Forest plot including one additional study on leisure-time physical activity and heart failure mortality (Williams, 2013), dose-response analysis

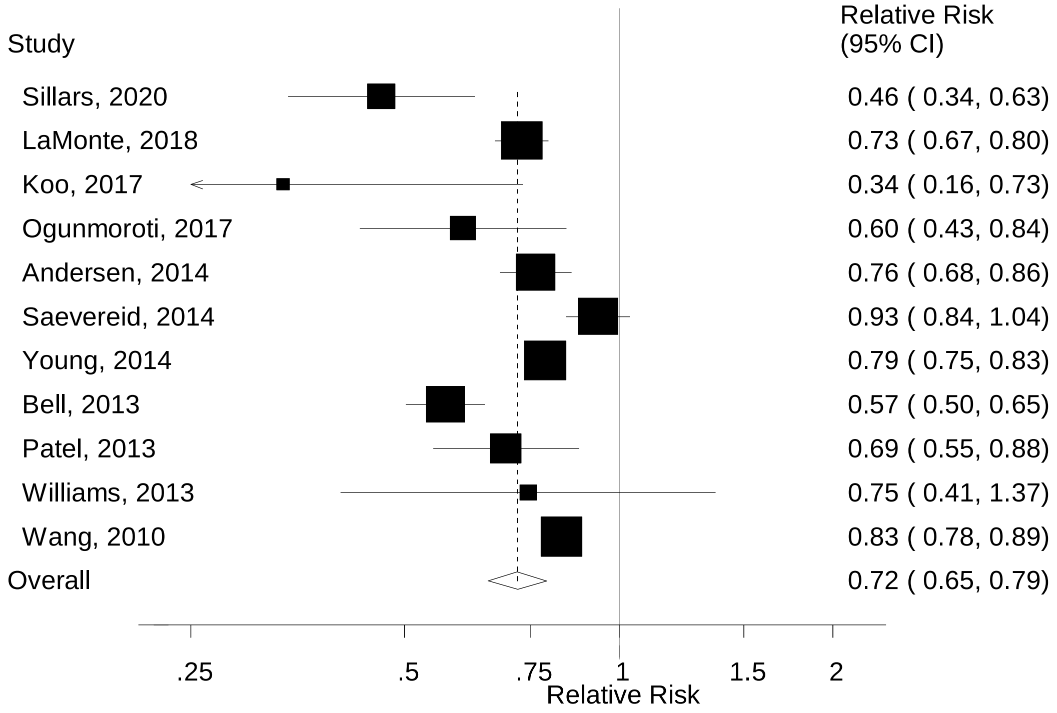

Supplementary Figure 11. Walking and heart failure, high vs. low analysis including one study on heart failure mortality (Williams, 2013)

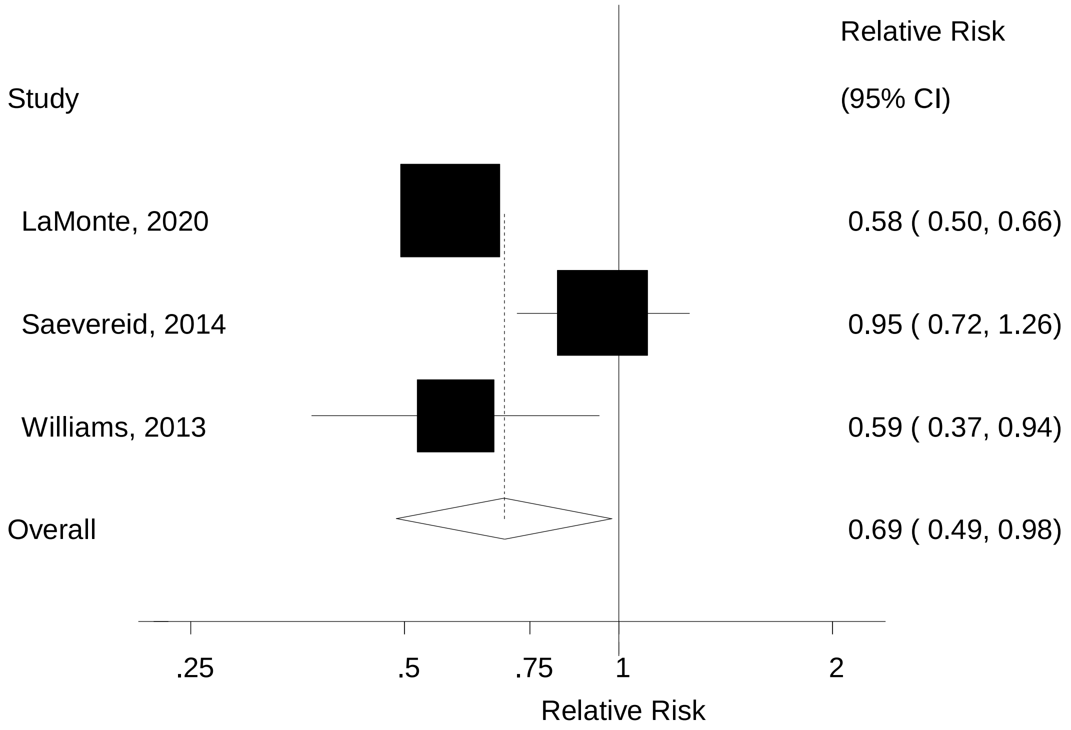

Supplementary Figure 12. Funnel plot of cardiorespiratory fitness and heart failure

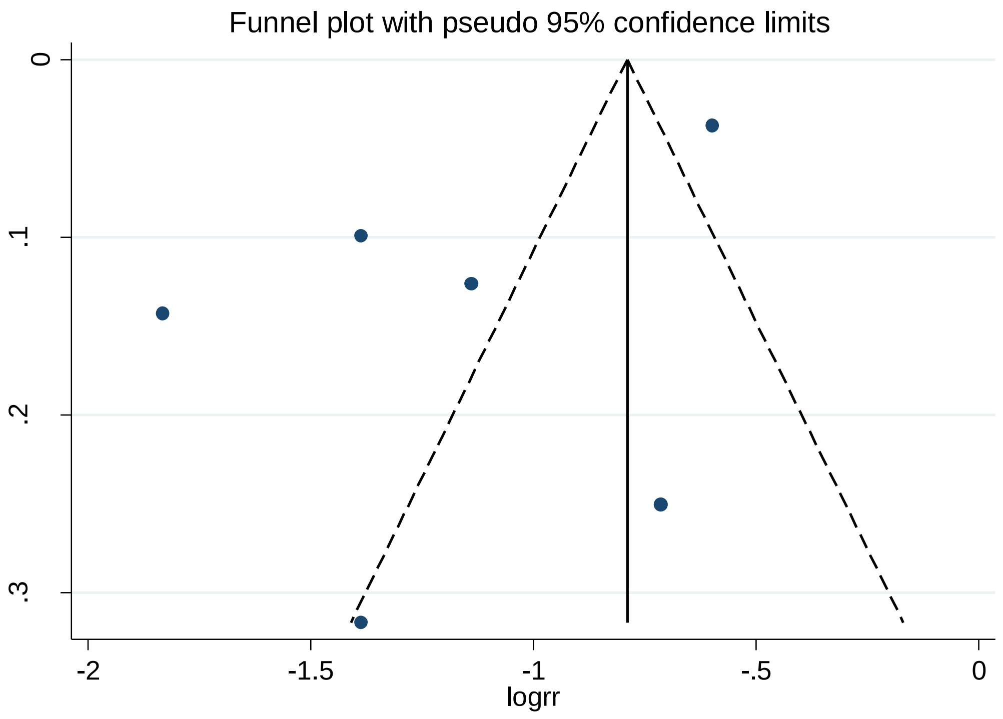

Supplementary Figure 13. Influence analysis of cardiorespiratory fitness and heart failure

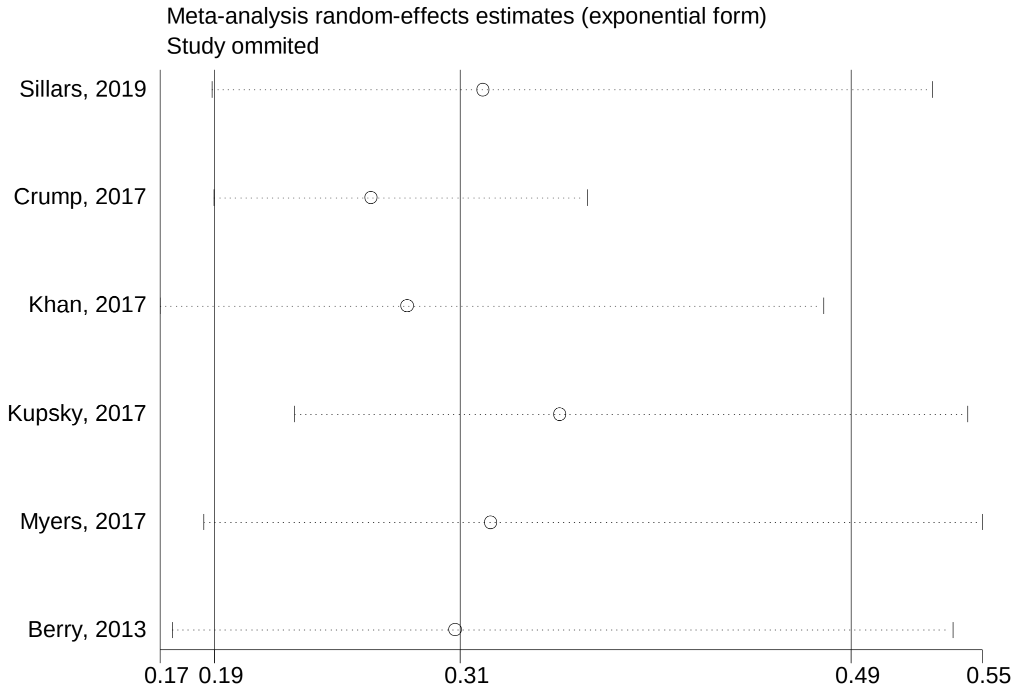

Study omitted	e^coef.	[95% Conf.	Interval]
Sillars, 2019	0.32027945	0.19334914	0.53053731
Crump, 2017	0.26780850	0.19427399	0.36917651
Khan, 2017	0.28463578	0.16893144	0.47958824
Kupsky, 2017	0.35624844	0.23200895	0.54701746
Myers, 2017	0.32391503	0.18943322	0.5538677
Berry, 2013	0.30708280	0.17459272	0.54011327
Combined	0.30946372	0.1945074	0.49236066

Supplementary Figure 14. Leisure time physical activity and heart failure, stratified by ethnicity, high vs. low analysis

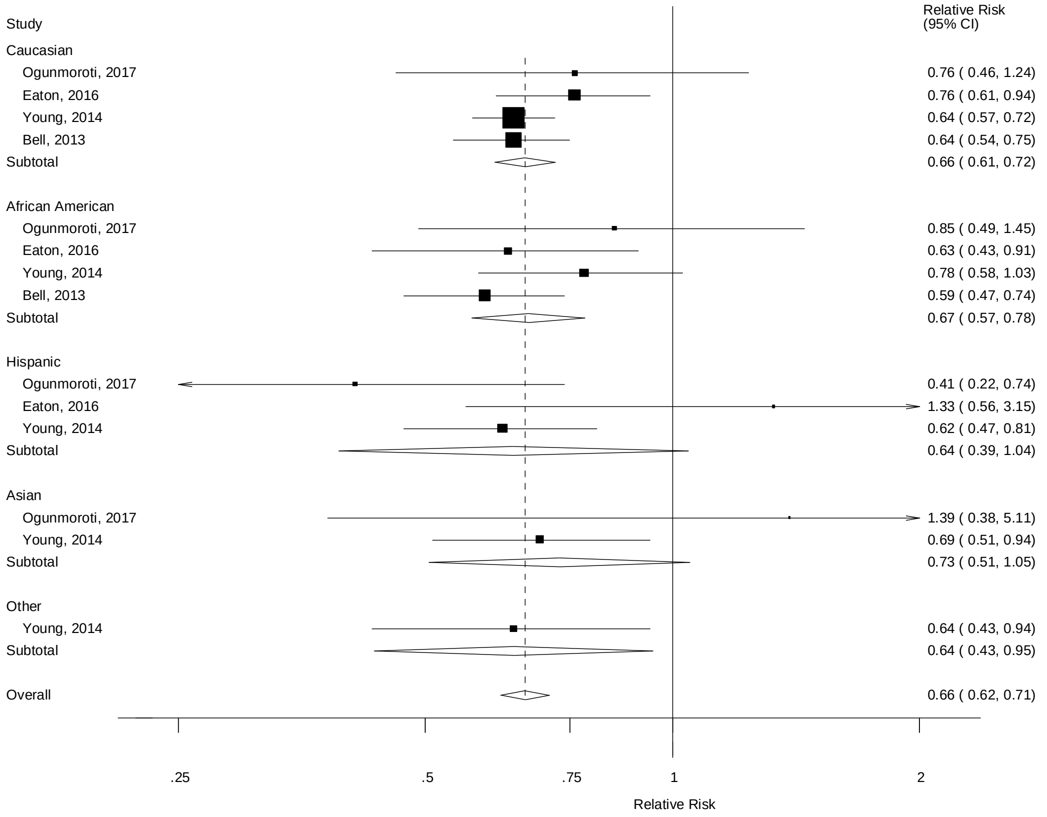

Supplementary Figure 15. Leisure-time physical activity and heart failure (per 20 MET-hours/week), stratified by whether conversions were made in estimating MET-hours/week (Converted) or whether MET-hours/week was reported directly (Not converted)

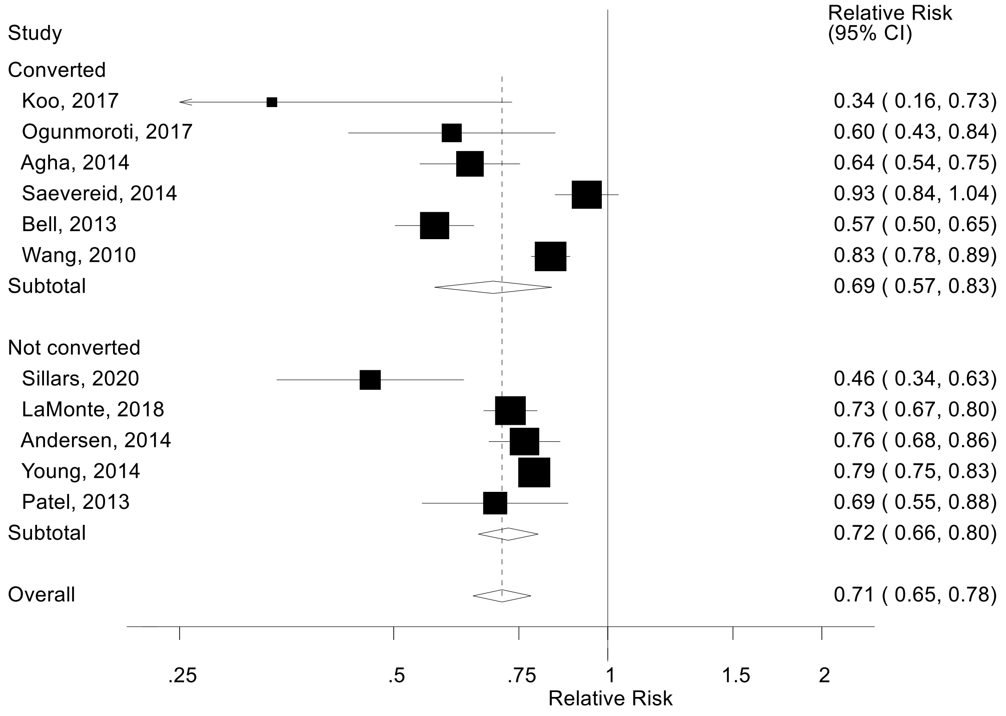

Supplement: Supplementary file 1 — Supplementary material 1 (PDF 426 kb) [file 10654_2020_693_MOESM1_ESM.pdf]
